# Anchoring of Heterochromatin to the Nuclear Lamina Reinforces Dosage Compensation-Mediated Gene Repression

**DOI:** 10.1371/journal.pgen.1006341

**Published:** 2016-09-30

**Authors:** Martha J. Snyder, Alyssa C. Lau, Elizabeth A. Brouhard, Michael B. Davis, Jianhao Jiang, Margarita H. Sifuentes, Györgyi Csankovszki

**Affiliations:** Department of Molecular, Cellular, and Developmental Biology, University of Michigan, Ann Arbor, Michigan, United States of America; Brown University, UNITED STATES

## Abstract

Higher order chromosome structure and nuclear architecture can have profound effects on gene regulation. We analyzed how compartmentalizing the genome by tethering heterochromatic regions to the nuclear lamina affects dosage compensation in the nematode *C*. *elegans*. In this organism, the dosage compensation complex (DCC) binds both X chromosomes of hermaphrodites to repress transcription two-fold, thus balancing gene expression between XX hermaphrodites and XO males. X chromosome structure is disrupted by mutations in DCC subunits. Using X chromosome paint fluorescence microscopy, we found that X chromosome structure and subnuclear localization are also disrupted when the mechanisms that anchor heterochromatin to the nuclear lamina are defective. Strikingly, the heterochromatic left end of the X chromosome is less affected than the gene-rich middle region, which lacks heterochromatic anchors. These changes in X chromosome structure and subnuclear localization are accompanied by small, but significant levels of derepression of X-linked genes as measured by RNA-seq, without any observable defects in DCC localization and DCC-mediated changes in histone modifications. We propose a model in which heterochromatic tethers on the left arm of the X cooperate with the DCC to compact and peripherally relocate the X chromosomes, contributing to gene repression.

## Author Summary

DNA isolated from the nucleus of a single human cell, if stretched out, would be 3 meters long. This amount of DNA must be packaged into a nucleus, which is orders of magnitude smaller. DNA of active genes tends to be loosely packed and localized internally within the nucleus, while DNA of inactive genes tends to be tightly packed and localized near the nuclear periphery. We studied the effects of DNA compaction and nuclear localization on gene expression levels using regulation of the X chromosomes in the nematode *Caenorhabditis elegans* as a model. In this organism, hermaphrodites have two X chromosomes, and males have only one. Genes on the two X chromosomes in hermaphrodites are expressed at half the level compared to the male X, such that the two Xs together express as much gene products as the single X in males. We found that silent regions at the left end of hermaphrodite X chromosomes are tethered to the nuclear periphery, and these tethers are used to build a compact chromosome structure. If this process is defective, gene expression levels are elevated, but less than two-fold. These results indicate that chromosome compaction and nuclear localization contribute to influencing gene expression levels, but other mechanisms must also contribute.

## Introduction

Expression of genes must be tightly regulated both spatially and temporarily to ensure normal development. While our understanding of gene regulation at the level of transcription factor binding and modulation of chromatin structure is supported by an abundance of data, the contribution of the spatial organization of the nucleus to regulation of gene expression is not well understood. Regulation of sex chromosome-linked gene expression in the process of dosage compensation provides an excellent model to dissect the influence of different gene regulatory mechanisms on chromosome-wide modulation of gene activity. In the nematode *C*. *elegans*, dosage compensation downregulates expression of genes on the otherwise highly expressed X chromosomes of hermaphrodites, such that transcript levels from the two hermaphrodite X chromosomes are brought down to match transcript levels from the single X in males [1, 2]. A complex of proteins called the dosage compensation complex (DCC) binds the length of both hermaphrodite X chromosomes to regulate transcription. The DCC contains a subcomplex, condensin I^DC^, which is homologous to condensin complexes in all eukaryotes responsible for compaction and segregation of chromosomes in mitosis and meiosis [3–5].

Although a number of studies in recent years uncovered molecular mechanisms of DCC action, how these alterations in X chromosome structure repress gene expression remains unknown. Consistent with a similarity to mitotic condensins, DCC binding leads to compaction of hermaphrodite X chromosomes in interphase [6, 7]. The DCC also remodels the X chromosomes into topologically associating domains (TADs) with more regular spacing and stronger boundaries than those found on autosomes [8]. At the level of chromatin organization, posttranslational modifications of histones are also altered in a DCC-dependent manner: monomethylation of histone H4 lysine 20 (H4K20me1) becomes enriched, and acetylation of histone H4 lysine 16 (H4K16ac) becomes depleted on dosage compensated Xs as compared to autosomes [9, 10]. Analysis of gene expression in H4K20 histone methyltransferase (HMT) mutants revealed that changes in H4K20me1 levels contribute to DCC-mediated repression, but are not fully responsible for the observed two-fold repression [11]. The relative contributions of chromosome condensation and partitioning of the chromosome into TADs are unclear. To date, no correlation has been found between genes being subjected to DCC-mediated repression and regions of the chromosome bound by the DCC [12, 13], DCC-induced changes in TADs [8] or posttranslational histone modifications [10]. These observations led to the suggestion that the DCC regulates gene expression not on a gene-by-gene basis, but rather in a chromosome-wide manner.

A model of DCC-mediated chromosome-wide repression is consistent with the idea of the formation of a repressive nuclear compartment. Organization of chromosomes within the nucleus is not random, but rather active and inactive portions of the genome are clustered together and separated into spatially distinct compartments [14–16]. One prominent feature of nuclear organization is positioning heterochromatic regions at the nuclear periphery or near the nucleolus [17–19]. An open question is to what extent this level of organization influences gene activity, rather than being a consequence of it. In this study we investigated the role of nuclear organization, particularly the tethering of heterochromatic regions to the nuclear lamina, in regulating genes on dosage compensated X chromosomes in *C*. *elegans*.

Genome-nuclear lamina interactions change dynamically during cellular differentiation and development and are known to influence gene activity. In *C*. *elegans*, tissue specific promoters are localized randomly in nuclei of undifferentiated cells, reflecting the pluripotent state of these cells. As cells commit to specific fates and differentiate, active promoters move toward the nuclear interior, while repressed promoters move toward the nuclear periphery [20]. Disruption of nuclear lamina anchoring by depletion of lamin (LMN-1) or lamin-interacting proteins leads to derepression of otherwise silent transgenes, demonstrating the relevance of the anchoring process to gene repression, at least in the context of transgenes [21]. Anchoring of these heterochromatic transgenic arrays to the nuclear lamina requires trimethylation of histone H3 lysine 9 (H3K9me3) by the HMTs MET-2 and SET-25, as well as the chromodomain protein CEC-4 [22, 23]. The relevance of this process to the regulation of endogenous gene expression is less clear. Gene expression does not change dramatically in the absence of H3K9me3 or CEC-4, but repression induced by heterochromatic anchoring does help restrict alternate cell fates in development [22, 23]. These observations indicate that likely multiple mechanisms contribute to repression of genes not expressed in a given cell type, and the contribution of lamina anchoring to gene regulation may only become apparent in sensitized backgrounds. Similar results were obtained in other organisms. For example, in differentiating mouse embryonic stem cells, genome-nuclear lamina interactions are remodeled such that some, but not all, genes move away from the nuclear lamina when activated [24].

Consistent with a generally repressive environment, regions of the genome associated with the nuclear lamina (lamina associated domains, or LADs) are depleted of active chromatin marks and are enriched for repressive marks such as H3K9 and H3K27 methylation in a variety of organisms [24–27]. These silencing marks, and the enzymes that deposit them, are required for peripheral localization of heterochromatic transgenes and some developmentally regulated endogenous sequences [23, 28–30]. Artificial tethering of genes to the nuclear lamina leads to repression of some, but not all, genes [31–34]. These observations are consistent with the idea that the vicinity of the nuclear lamina is a repressive environment, yet it is not incompatible with transcription. Therefore, subnuclear compartmentalization may not be a primary driver of gene expression levels, but rather serve as a mechanism to stabilize existing transcriptional programs [22].

Here we show that anchoring of heterochromatic regions to the nuclear lamina contributes to shaping the higher order structure and nuclear localization of dosage-compensated X chromosomes. These X chromosome-specific phenotypes were observed in multiple tissues, and thus appear to be inherent to the chromosome and not any cell-type specific differentiation program. We show that heterochromatin integrity and its nuclear lamina anchors are required for spatial organization of the nucleus and dosage compensation mediated condensation of the X chromosome. In mutant strains that lack these anchors, despite normal DCC localization to the X chromosome, we observe a small, but significant level of X derepression, consistent with the idea that anchoring contributes to stabilizing gene repression. Remarkably, tethering of heterochromatic regions of the X chromosome to the nuclear lamina affects the entire chromosome, not only the tethered domain. We propose a model in which the tethered domain nucleates formation of a peripherally localized compact structure, which facilitates the action of the DCC to compact the entire X chromosome.

## Results

**Fig 1:**
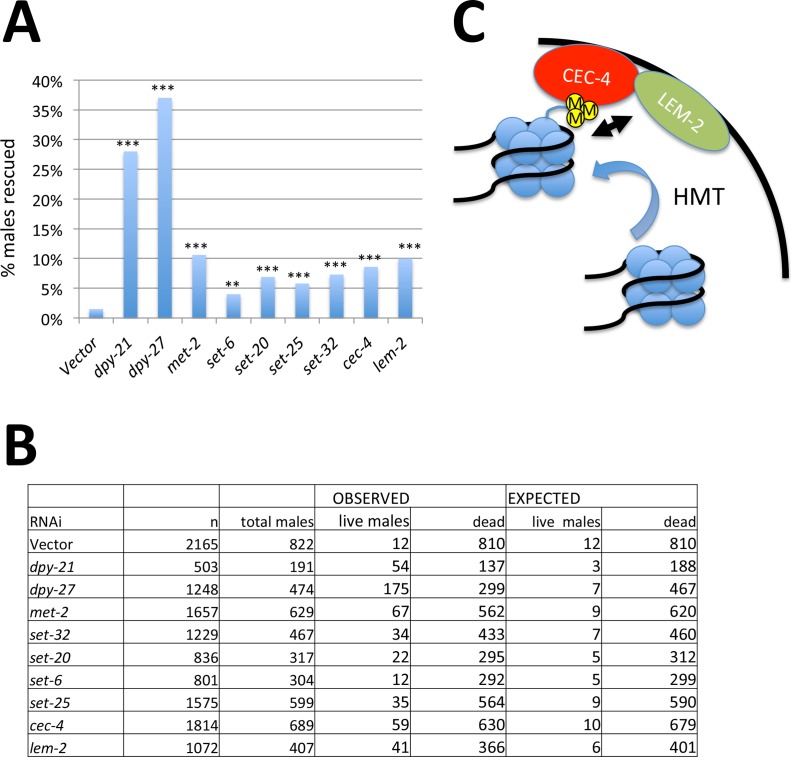
RNAi screen to identify genes that promote dosage compensation. **(A)** Male rescue assay. RNAi-mediated depletion of the indicated genes in the *him-8 xol-1 sex-1* background led to rescue of the indicated percentage of males. Depleting DCC components DPY-21 and DPY-27 rescues a larger percentage of males than depletion of the other genes identified in this screen Asterisks indicate statistical significance based on Chi square test analysis of results, with expected rescue being equivalent to vector RNAi. * = p<0.05, ** = p<0.01, *** = p<0.001. **(B)** Raw data and expected table used in Chi square analysis. **(C)** Proposed mechanism of anchoring heterochromatic regions to the nuclear lamina. HMTs methylate H3K9. The chromodomain protein CEC-4 binds to this chromatin mark. Bound genomic regions are enriched for interactions with the nuclear lamina protein LEM-2.

In order to identify chromatin modifying genes that influence dosage compensation, we previously performed a targeted RNAi screen to analyze genes implicated in chromatin regulation, including histone variants, as well as genes containing chromo, bromo, or set domains [35]. The assay is based on rescue of males that inappropriately turn on dosage compensation. The DCC assembles on the X chromosome of *xol-1(y9) sex-1(y263)* males, leading to insufficient expression of genes from the single X chromosome and thus lethality. RNAi-mediated disruption of dosage compensation can rescue a proportion of these males. Control vector RNAi leads to background level of rescue (about 1.5%), while RNAi of a component of the DCC rescues over 25% of males. We previously described the screen in detail, as well as the role of one of the hits from the screen, the histone H2A variant HTZ-1 [35]. In this study we characterize the remaining genes identified in this screen that led to low but reproducible levels of male rescue. These genes include the histone methyltransferases *met-2*, *set-32*, *set-20*, *set-6*, *set-25*, and the chromodomain protein *cec-4* (Fig 1). All of these histone methyltransferases are known (*met-2*, *set-25*, [23]) or predicted (*set-6*, *set-20*, *set-32* [36]) to modify H3K9. H3K9 methylation and the chromodomain protein CEC-4 were previously shown to work together in regulating nuclear organization and anchoring heterochromatic transgenic arrays to the nuclear lamina [21, 22, 37]. We therefore included in our analysis LEM-2 (hMAN1), a non-essential component of the nuclear lamina. RNAi of the single *C*. *elegans* lamin gene LMN-1 leads to embryonic lethality [38], precluding this type of analysis. However, RNAi-depletion of LEM-2 led to male rescue comparable to, or higher than, the rescue caused by depletion of the HMTs or CEC-4 (Fig 1). Chi square test of the data indicated that all genes rescued significantly more males than vector RNAi (Fig 1B). To ensure that the rescue is reproducible, we also performed the rescue assay with a subset of the identified genes in four independent biological replicates and analyzed the results using Student's t-test. In this analysis, all genes identified in the screen with the exception of *set-6* and *set-20* rescued significantly more males than vector RNAi (S1 Fig).

### X chromosome decondensation in mutants

**Fig 2:**
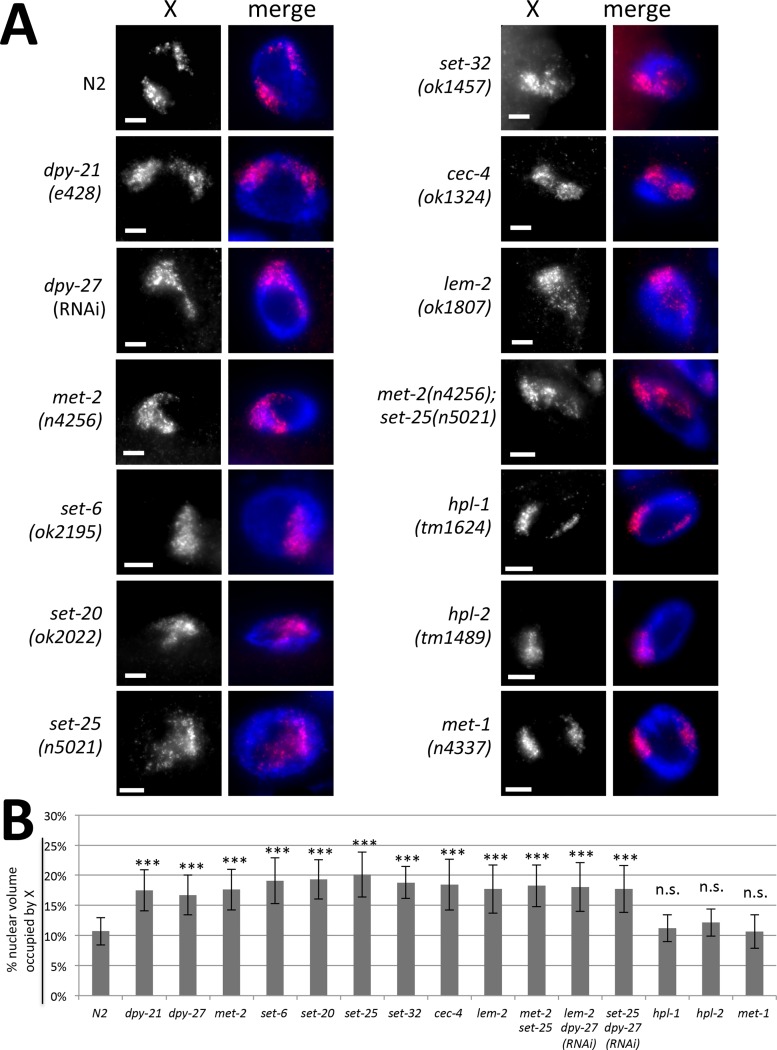
X chromosome decondensation in mutants. **(A)** X chromosome paint FISH (red) in representative images of intestinal nuclei (DAPI, blue) of hermaphrodite adult worms in each genotype. The X chromosomes are compact and peripherally localized in wild type (N2), *hpl-1*, *hpl-2* and *met-1* mutant hermaphrodites, but are decondensed and more centrally located in the other mutants. Scale bar, 5 μm. **(B)** Quantification of X chromosome volumes normalized to nuclear size (n = 20 nuclei). Error bars indicate standard deviation. n.s = p>0.05 not significant; *** = p<0.001 by Student's t-test (N2 compared to appropriate mutant).

The finding of H3K9 methyltransferases, CEC-4, and LEM-2, in this screen suggested that nuclear organization, and specifically anchoring of chromosomal regions to the nuclear lamina (Fig 1C), might affect dosage compensation. To investigate X chromosome morphology and its location in the nucleus in the absence of these proteins, we performed X chromosome paint fluorescence *in situ* hybridization (FISH) in the various mutant backgrounds. First we investigated the 32-ploid nuclei of the intestine, because their large size facilitates visualization of chromosome territories. In wild type (N2) hermaphrodite worms, the X chromosome territories are kept compact by the action of the DCC [39] and the territory is found near the nuclear lamina (Fig 2A). Visual inspection of the X chromosome territories in *met-2(n4256)*, *set-6(ok2195)*, *set-20(ok2022)*, *set-25(n5021)*, *set-32(ok1457)*, *cec-4(ok1324)*, and *lem-2(ok1807)* hermaphrodites revealed that the nuclear territory occupied by the X chromosomes became larger. As a control, we also analyzed the X chromosomes in *met-1(n4337)*, *hpl-1(tm1624)* and *hpl-2(tm1489)* mutants. MET-1 is an unrelated HMT, while HPL-1 and HPL-2 are homologs of the highly conserved heterochromatin protein and H3K9me3 binding protein HP-1 [40] (Fig 2A). To quantify X chromosome condensation, we measured the volumes of X chromosome territories, as in [39]. Briefly, we generated intensity threshold-based 3D masks for the X chromosome (X paint signal) and for the nucleus (DAPI signal). We then calculated the volume of the X chromosome and of the nucleus, and determined the portion of the nucleus occupied by the X chromosome. Normalization to total nuclear volume was necessary due to the large variability in nuclear size after the harsh treatments involved in FISH. Quantification of the volume of the X chromosome territory showed that in the H3K9 HMT mutants, as well as in *cec-4* and *lem-2* mutants, the X chromosome occupied a much larger portion of the nucleus than in control wild type, or *met-1*, *hpl-1* or *hpl-2* mutant hermaphrodites. Lack of X chromosome condensation defects in *hpl-1* and *hpl-2* mutants are consistent with a previous study that reported no defects in nuclear lamina anchoring of heterochromatic transgenic arrays in *hpl-1* or *hpl-2* mutants [22]. In nuclei of wild type worms the X chromosome occupied about 10% of the nuclear volume, compared to an average of up to 20% percent in mutants (p<0.001, Student's t-test, for all comparisons between a mutant and wild type) (Fig 2B). In fact, the degree of decondensation in *set-25(n5021)* mutants is even larger than in DCC mutant or RNAi-depleted hermaphrodites (*dpy-21(e428)* and *dpy-27(RNAi)* [39]) (p = 0.0251 for comparison with *dpy-21*, and p = 0.00442 for comparison with *dpy-27;* other differences were not statistically significant) (Fig 2). We conclude that the X chromosome is decondensed to a significant degree in worms carrying mutations in DCC subunits, as well as in H3K9 HMT, *cec-4* and *lem-2* mutants.

SET-25 and MET-2 are the only well characterized HMTs among the ones we identified. MET-2 introduces H3K9 mono- and dimethylation, while SET-25 introduces H3K9 trimethylation. Complete lack of H3K9 methylation, and loss of anchoring of heterochromatic arrays, are only observed in the *met-2 set-25* double mutants and not in *set-25* or *met-2* single mutants [23]. We therefore analyzed X chromosome structure in the *met-2(n4256) set-25(n5021)* double mutant strain and found that the X chromosome morphology is comparable to single mutants without an obvious additive effect (p = 0.56 for *met-2* compared to *met-2 set-25*; p = 0.11 for *set-25* compared to *met-2 set-25*) (Fig 2A and 2B). For the rest of this study we concentrated on *lem-2*, *set-25 or met-2 set-25*, *and cec-4* mutants, and we will refer to them collectively as “tethering mutants”.

One possible explanation for X decondensation phenotype is that the tethering defects diminish the ability of the DCC to condense the X chromosome. For example, the DCC may use these heterochromatic tethers as nucleation sites for a more compact chromosomal organization. An alternative possibility is that lack of tethering leads to chromosome decondensation independent of the DCC. We tested whether simultaneous disruptions of tethering and the DCC lead to increased levels of decondensation by measuring X chromosome volumes in *set-25* and *lem-2* mutants that were depleted of DPY-27 using RNAi (Fig 2B). X chromosomes of nuclei in *dpy-27(RNAi*) treated *lem-2* mutants were significantly different from wild type, but statistically indistinguishable from either *lem-2* mutants (p = 0.77, Student's t-test) or *dpy-27(RNAi)* (p = 0.26). Similarly, X chromosomes of nuclei in *dpy-27(RNAi)* treated *set-25* mutants were significantly different from wild type, but statistically indistinguishable from *set-25* mutants (p = 0.052) and *dpy-27(RNAi)* (p = 0.39). Therefore, at this resolution, we cannot detect any additional defects when tethering mutations are combined with DCC depletion, consistent with the hypothesis that the DCC and tethering genes work together, and are both required, to condense the X chromosomes.

To determine whether the phenotype is specific to the 32-ploid intestinal nuclei, we also examined diploid tail tip hypodermal cells hyp 8–11. Results were comparable to intestinal cells. In wild type cells, the X chromosome occupies about 10% of the nucleus, while it occupies a much larger portion of the nucleus in anchoring mutants (p<0.001 for all mutant comparisons to wild type) (S2 Fig).

### The dosage compensated X chromosome relocates to a more central position in tethering mutants

**Fig 3:**
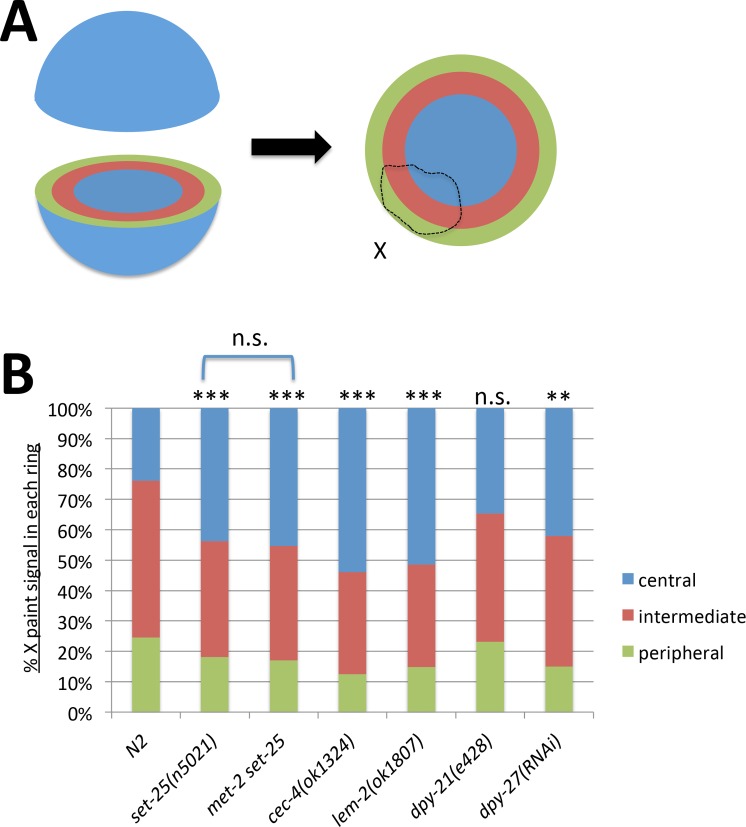
The X chromosome relocates centrally in the nucleus. **(A)** A diagram of the three-zone assay. An optical section from the middle of the nucleus was divided into three concentric rings of equal area. The proportion of the X chromosome paint signal in each zone (peripheral-intermediate-central) was quantified. **(B)** Results of quantification of the three-zone assay using whole X paint FISH probes in hermaphrodite intestinal nuclei (n = 10). In tethering mutants, a larger portion of the X chromosome is located in the central zone compared to wild type hermaphrodites. Relocation to a central region is less significant in DCC mutants or DCC-depleted hermaphrodites. Asterisks indicate statistical analysis (Student's t-test) of the centrally located portion of the X chromosome (shown in blue). n.s. = p>0.05, * = p<0.05, ** = p<0.01, *** = p<0.001. See S1 Table for statistical data.

Previous studies showed that tethering mutants have a defect in anchoring heterochromatic transgenic arrays to the nuclear lamina [21–23]. Similarly, visual inspection of our images suggested that tethering mutants have a defect in subnuclear positioning of the X chromosome resulting in the X occupying a more central position (Fig 2). To quantify this defect, we performed an analysis similar to the three-zone assay used in [20]. We selected nuclei that were spherical or ellipsoid shaped. From the Z-stacks generated during imaging, we selected the optical section toward the middle of the nucleus with the largest and brightest X-paint signal. This optical section was divided into three-zones of equal area, and the portion of the X signal located in each zone was quantified (Fig 3A). The percentage of nuclei in each genotype that can be quantified using this assay is shown in S3 Fig. Representative irregularly shaped nuclei are also shown to illustrate that the X chromosome appeared qualitatively similar to the X chromosomes in round or ellipsoid shaped nuclei: compact and peripherally located in N2 hermaphrodites, and larger and more centrally located in tethering mutants. The three-zone assay showed that in wild type (N2) nuclei only about 20% of the X chromosome signal was located in the central zone, while in tethering mutants over 40% of the X signal was located in this zone, suggesting that the X chromosome relocates to a more central position within the nucleus (Fig 3B). Comparisons of the portions of the X chromosome located in the central zone revealed statistically significant differences in all tethering mutants. As for volume measurement, the three-zone assay again failed to reveal additional defects in *met-2 set-25* double mutants compared to *set-25* single mutants. We then compared this effect to mutating or depleting a subunit of the DCC by RNAi. The three-zone assay showed less significant relocation of the X toward the center in *dpy-27* RNAi-treated hermaphrodites compared to tethering mutants. In *dpy-21* mutants, although the portion of the X in the central zone increased from 24% to 34%, the difference did not reach statistical significance (Fig 3B). One possible reason for the less significant relocation in dosage compensation mutants is the fact that *dpy-27(RNAi)* or a mutation in *dpy-21* does not completely disrupt dosage compensation function. Complete lack of DCC activity would be lethal to hermaphrodites, precluding this type of analysis (see analysis of the male X below).

**Fig 4:**
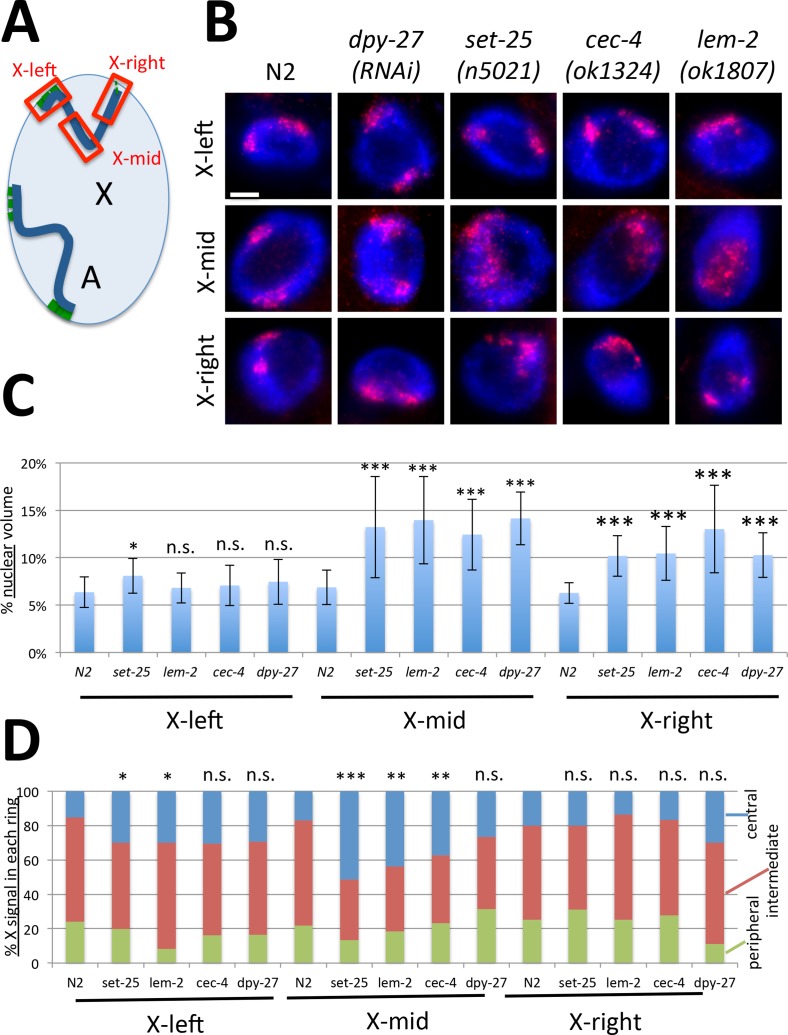
The middle region of the X chromosome is most affected in the absence of heterochromatic tethers. **(A)** Autosomes are anchored to the nuclear lamina at both chromosome arms (anchors shown in green), while the X chromosome only has a significant anchored domain at the left end. Probes used in FISH analysis are indicated in red. Each probe covered an approximately 3–4 Mb genomic region. **(B)** Representative images of X-left, X-mid, X-right FISH analysis in each genotype. The mid-X region appears most decondensed and most centrally located in mutants. Scale bar, 5 μm. **(C)** Quantification of volumes occupied by the indicated FISH probes, normalized to nuclear size (n = 12 nuclei). Error bars indicate standard deviation. The greatest degree of decondensation in mutants is observed for the mid-X probe. **(D)** Three zone assay for each probe (n = 12 nuclei). The greatest degree of central relocation is observed for the mid-X probe. Asterisks indicate statistical analysis of mutant to wild type comparisons of volumes in (C) and centrally located portion of the X in (D) using Student's t-test. n.s. = p>0.05, * = p<0.05, ** = p<0.01, *** = p<0.001. See S1 Table for statistical data.

H3K9me3 is generally found in heterochromatic regions of the genome. In *C*. *elegans*, several megabase regions at both ends of autosomes and the left end of the X chromosome are enriched for this mark [41]. These H3K9me3-enriched domains also coincide with nuclear lamina-associated domains, as assessed by ChIP [26] or DamID [23]. Together these results suggest a model in which both arms of autosomes and the left of arm of the X chromosome are tethered to the nuclear lamina [23, 26, 42–44] (Fig 4A). Peripheral localization of heterochromatic chromosomal regions may be mediated by CEC-4, as is the case for heterochromatic transgenes [22].

To examine whether heterochromatic segments of the X chromosomes are affected differently than other chromosomal regions, we prepared probes to approximately 3–4 Mb regions of the chromosome. The X-left probe covers a region enriched for H3K9me3 and LEM-2, the X-mid probe covers a gene-rich portion of the chromosome with very little H3K9me3 and LEM-2, and the X-right probe covers a region with intermediate levels of H3K9me3 and LEM-2 (Fig 4A and 4B). We then assessed the level of decondensation of each of these regions by measuring the proportion of the nuclear volume occupied by this region of the X chromosome (Fig 4C). Surprisingly, the left end of the X chromosome was least affected and remained condensed both in tethering mutants and in DCC-depleted hermaphrodites. We only observed a mild level of decondensation in *set-25* mutants. By contrast, the gene-rich middle portion of the chromosome was most affected and was significantly decondensed in all tethering mutants. The right end of the chromosome, which contains some LEM-2 and H3K9me3 peaks, but fewer than the left end, exhibited an intermediate phenotype.

The gene-rich middle portion of the X chromosome was not only decondensed but also appeared to exhibit the greatest degree of central relocation. To quantify this effect, we again performed the three-zone assay and found that indeed the mid-X region was most affected (Fig 4D). While on average only 17% of the X-mid probe was located in the central zone in wild type nuclei, up to 50% of the same region was found in this zone in tethering mutants. Relocation to a central position was less obvious for the left end of the chromosome and was not detectable for the right end. These results were at first unexpected. However, they are consistent with previous observations that have hinted at the existence of redundant tethering mechanisms in differentiated cells. The tethering mechanism mediated by heterochromatin is only essential for anchoring of heterochromatic arrays in embryonic cells, and the arrays remain anchored in differentiated tissues even in the absence of SET-25 and MET-2 [23]. Similarly, CEC-4 is required for anchoring in embryos, but other, yet unknown, mechanisms can compensate for the lack of CEC-4 protein in differentiated cells [22]. We note that all of our analyses were performed in terminally differentiated postmitotic cells of adult animals. Our results suggest that regions of the left end of the X chromosome are anchored to the nuclear periphery by an additional mechanism that is independent of SET-25, CEC-4, and LEM-2, and loss of H3K9me3-lamina mediated anchoring mechanism is not sufficient to significantly relocate this region. The lack of heterochromatic anchoring mechanism affects the middle of the chromosome disproportionately, even though this region is depleted of H3K9me3 and LEM-2 interactions. One possible interpretation of this result is that the few H3K9me3 sites and LEM-2-bound regions present in the middle of the chromosome represent the only anchoring mechanism present in this region. In the absence of these tethers, the mid-X region is free to relocate more centrally, while redundant anchors maintain tethering to a greater degree at the two chromosome ends. The other interpretation, which is not mutually exclusive, is that heterochromatic anchors at the left end of the chromosome are used to nucleate a compact structure, which is required to be able to pull the rest of the X chromosome toward the periphery and compact it efficiently (see Discussion).

Defects in DCC function had a somewhat different effect. The mid-X region was more decondensed after *dpy-27(RNAi)* than the right end, and the left end was unaffected, similar to the decondensation defects seen in tethering mutants. Less significant decompaction of the left end may be related to the somewhat lower levels of DCC binding in this region [41, 45]. Alternatively, nuclear lamina tethers at the left end may be sufficient to compact this region even in the absence of the DCC. Although the portion of the mid-X region in the central domain increased in *dpy-27* RNAi, the difference did not reach statistical significance, suggesting again insufficient disruption of dosage compensation (Fig 4C and 4D).

### Chromosomal phenotypes are dosage compensation dependent

**Fig 5:**
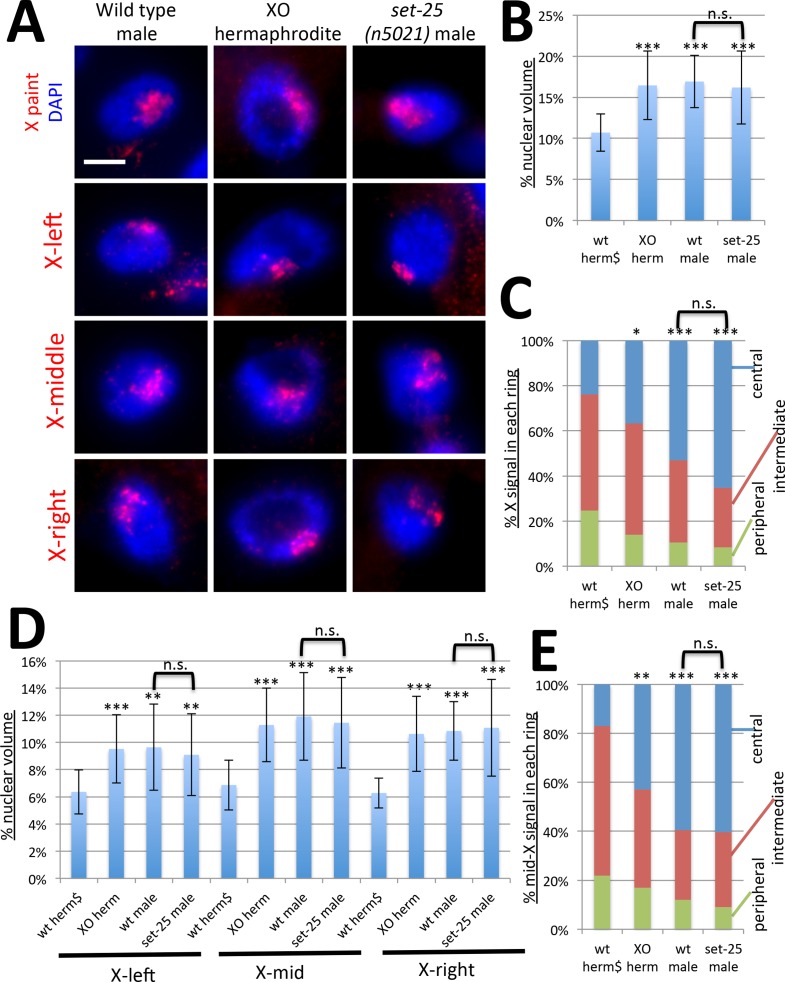
The X chromosome is decondensed and centrally located in the absence of dosage compensation in XO animals. **(A)** Chromosome paint FISH (red) in intestinal nuclei (DAPI, blue) of male adult worms, XO hermaphrodites and *set-25* mutant males using whole X paint probe, and probes to the left, middle, and right domains of the X chromosome. The X chromosome, and the middle region of the X chromosome, appear large and diffuse and are located more toward the nuclear interior. *set-25* mutations do not have additional effects on X chromosome morphology in males. Scale bar, 5 μm. **(B)** Quantification of volumes occupied by the X paint probe (n = 20 nuclei). The wild type hermaphrodite data point (wt herm) is repeated from Fig 2B and is marked by $ sign. **(C)** Three-zone assay for the whole chromosome X paint probe (n = 10). Wt herm data point is repeated from Fig 3B ($). **(D)** Quantification of volumes occupied by the X-left, X-mid, and X-right probes normalized to nuclear size (n = 20). Wt herm data points are repeated from Fig 4C ($). **(E)** Three zone assay for the mid-X probe (n = 10). Wt herm data point is repeated from Fig 4D ($). Error bars in (B) and (D) indicate standard deviation. Asterisks indicate statistical analysis compared to wild type hermaphrodites for volumes in (B) and (D) and centrally located portion of the X or mid-X (C) and (E), using Student's t-test. wt male and *set-25* male comparisons are also shown as indicated. n.s. = p>0.05, * = p<0.05, ** = p<0.01, *** = p<0.001. See S1 Table for statistical data.

To examine these X chromosomal phenotypes in the complete absence of DCC activity, we analyzed the X chromosome in males and in XO animals that develop as hermaphrodites due to a mutation in the *her-1* gene required for male development [46] (Fig 5). The XO hermaphrodites also carry a null mutation in the dosage compensation gene *sdc-2* to ensure that all XX progeny die due to dosage compensation defects and only XO animals survive [47]. The DCC is XX hermaphrodite-specific and does not bind to the male X or the X chromosome in XO hermaphrodites, therefore these backgrounds allow us to examine X chromosome structure in the complete absence of the DCC, but in the presence of heterochromatic anchors. In wild type males and in XO hermaphrodites, the single X occupied an large proportion of the nucleus, about 16%, as we previously observed, which is significantly different from the 10% seen in wild type hermaphrodites [39] (Fig 5A and 5B). It is also different from what was seen previously in nuclei of young embryos, possibly due to the differences in stage of development and differentiation status [7]. Note that the level of decondensation in males and XO hermaphrodites is greater than in tethering mutants. In XO animals, the single X chromosome occupies 16% of the nucleus, compared to the two Xs occupying 18–20% in tethering mutant hermaphrodites. However, in *set-25* mutant males, the X did not decondense further compared to normal males (Fig 5A and 5B). In addition, the three-zone assay revealed that the X chromosome is located significantly more centrally in XO hermaphrodites and males, compared to wild type hermaphrodites (Fig 5C). Irregularly shaped nuclei that cannot be quantified using this assay also appeared to have large centrally located X chromosomes (S3 Fig). While in *set-25* mutant males a slightly higher proportion of the X chromosome was located in the central zone, this difference was not statistically significant (Fig 5C). These results indicate that the activity of the DCC is required to condense and peripherally relocate the X chromosome, and that the lack of both DCC function and heterochromatic tethers (in *set-25* males) does not lead to additional defects.

We next analyzed the left, middle and right regions of the X chromosome in XO animals (Fig 5A, 5D and 5E). All regions of the X chromosome were decondensed in XO animals, compared to hermaphrodites, but mutations in *set-25* did not lead to any further decondensation (Fig 5D). Furthermore, the mid-X region was more centrally located in XO animals than in hermaphrodites, but again mutations in *set-25* did not lead to additional central relocation. While we cannot exclude the possibility that the X chromosome is affected in tethering mutant males, we conclude that hermaphrodite X chromosomes are more severely affected by these mutations than male X chromosomes.

### Chromosomal phenotypes are X specific

**Fig 6:**
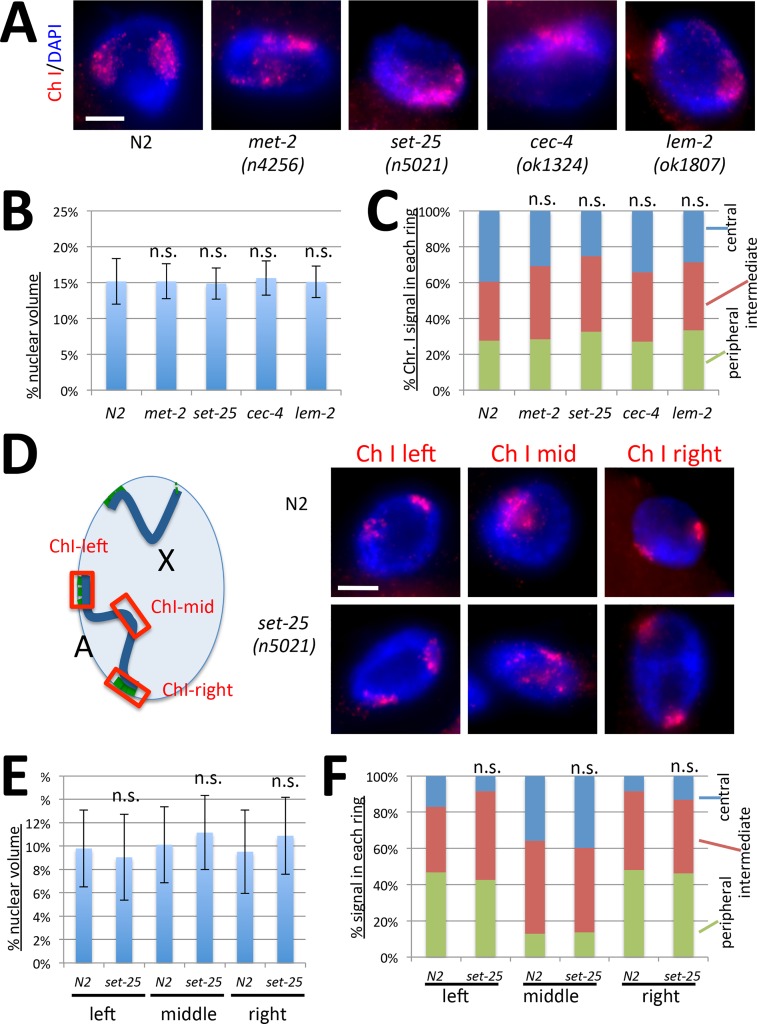
Chromosome I structure and organization is not affected in tethering mutants. **(A)** Chromosome I paint FISH (red) in representative images of intestinal nuclei (DAPI, blue) of hermaphrodite adult worms. Chromosome I appears comparably sized in each background. Scale bar, 5 μm. **(B)** Quantification of chromosome I volumes normalized to nuclear size (n = 12 nuclei). Error bars indicate standard deviation. **(C)** Three-zone assay for whole Chr I paint (n = 10 nuclei). The chromosome did not relocate to a more central position in any of the mutants (**D)** FISH analysis of the left, middle and right regions of Chr I in wild type (N2) and *set-25(n5021)* mutant hermaphrodites. Diagram (left) indicates locations of probes, representative images are shown on the right. The left and right ends of the chromosome are peripherally located, but the middle appears more centrally located in both backgrounds. **(E)** Quantification of volumes occupied by Chr I domains (n = 20 nuclei). Error bars indicate standard deviation. **(F)** Three-zone assay for the left, middle and right domains of Chr I (n = 10 nuclei). The middle domain is more centrally located than the left and right arms in both genotypes. Student's t-test did not reveal any statistically significant differences for volume measurements in (B) and (E), or for the portion of chromosome located in the central zone in (C) and (F), mutant compared to wild type. n.s. = p > 0.05. See S2 Table for statistical data.

To determine whether the chromosomal phenotypes are specific to the X chromosome, we analyzed the structure and localization of a similarly sized autosome, chromosome I (Fig 6A). Chromosome I occupies about 15% of the nucleus in wild type hermaphrodites, closely correlated with its genome content, indicating lack of condensation beyond genomic average [39]. As we found previously for dosage compensation mutants [39], the volume of chromosome I appeared unaffected in tethering mutants (Fig 6B). In addition, the three-zone assay revealed that a significant portion of chromosome I signal is located in the central zone in wild type hermaphrodites (39%) (Fig 6C). This value is significantly different from the value obtained for the X chromosome in wild type hermaphrodites (23%, Fig 3, p = 0.035, Student's t-test), and more similar to the X chromosome in tethering mutants (ranging from 43% to 55%, Fig 3). In addition, mutations in tethering mutants did not lead to any further central relocation of chromosome I compared to the same chromosome in wild type hermaphrodites (Fig 6C). These results suggest that the X chromosome is more sensitive to the loss of heterochromatic tethers than the autosomes.

To confirm these results, we further examined different domains of chromosome I (Fig 6D). Chromosome I has two anchored heterochromatic domains, one at each end (left and right), while the middle region lacks significant interactions with the nuclear lamina [23, 26, 41]. Our FISH analysis is consistent with these earlier observations. The left and right domains of the chromosome were located near the nuclear periphery, while the middle region was more centrally located. Neither volume measurements (Fig 6E), nor the three-zone analysis (Fig 6F) showed any significant differences between wild type (N2) and *set-25* mutant hermaphrodites. A significantly greater portion of the chromosome I middle domain was located in the central zone (36%) in wild type hermaphrodites compared to the X chromosome (17%, Fig 4, p = 0.018, Student's t-test), and this value was more comparable to the centrally located portion of the mid-X region in tethering mutants (ranging from 37% to 51%, Fig 4). These results indicate that in wild type hermaphrodites, the two ends of chromosome I are peripherally located, while the middle domain is more centrally located. Furthermore, we conclude that this organization does not change significantly in the absence of heterochromatic tethers, and that the observed chromosomal phenotypes are specific to the dosage compensated X chromosome.

### The distribution of H3K9me3 within the nucleus

**Fig 7:**
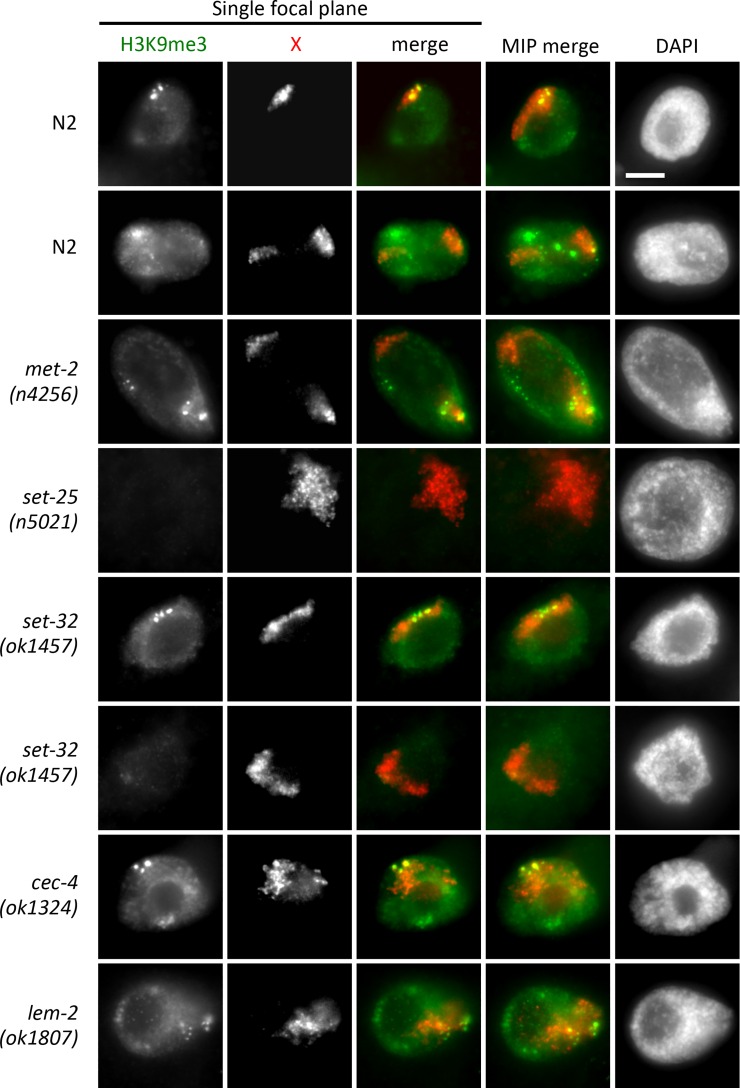
Analysis of H3K9me3 levels. Immunofluorescence analysis with antibodies specific to H3K9me3 (green), combined with antibodies specific to DCC subunit CAPG-1 (red) to mark the location of the X chromosomes. To illustrate the spatial proximity of the bright H3K9me3 foci to the X territory, single focal planes are shown. Maximum intensity projections of whole nuclei are shown for reference (right, MIP). The H3K9me3 signal is distributed diffusely in the nucleus with some peripherally localized bright foci. H3K9me3 signal intensity is only affected in *set-25* and *set-32* mutants. Scale bar, 5 μm.

Previous ChIP-chip analysis showed that H3K9me3 is enriched at both ends of autosomes and at the left end of the X chromosome, although peaks can be found elsewhere on the X as well [41]. To determine how this signal is distributed in the nucleus, we performed immunofluorescence microscopy (IF) with H3K9me3 specific antibodies in wild type cells and in tethering mutants (Fig 7). Antibodies specific to DCC subunit CAPG-1 were used as staining controls and to mark the territories of the X chromosomes. S4 Fig shows specificity of this newly developed antibody to CAPG-1. In wild type cells, the H3K9me3 signal was distributed all over the nucleus, with no obvious enrichment at the nuclear periphery, except for the presence of some peripherally located bright foci. Both the overall staining and the bright foci are H3K9me3-specific, as they were absent in *set-25* mutants. Sites of exceptionally high levels of H3K9me3 signal were not observed by ChIP [41]. Therefore, we interpret these bright foci as three-dimensional clustering of multiple H3K9me3 enriched loci. The X chromosome territory almost always contained, or was directly juxtaposed to one of these bright foci (Fig 7, top row). In rare cases, the X was not associated with the brightest foci, but foci of lesser intensity were still visible in the X territory (Fig 7, second row). H3K9me3 staining was comparable to wild type in *cec-4* and *lem-2* mutants, suggesting that the defects in tethering in these mutants are not related to lack of H3K9me3.

Notably, H3K9me3 was not absent in *met-2* mutants. In fact, *met-2* mutants were indistinguishable from wild type. This is in contrast to what was previously observed in *met-2* mutant embryos, where H3K9me3 levels were greatly reduced [23]. However, it is similar to what was observed in the germline, where *met-2* was reported to be dispensable for H3K9me3 [48], and similar to what we reported previously in intestinal nuclei of *met-2* mutants [49]. These results suggest tissue specific differences in the use of HMTs to deposit H3K9me3. Despite near-normal levels and distribution of H3K9me3 in *met-2* mutants, the X chromosomes were decondensed, suggesting that *met-2* contributes to the regulation of X chromosome structure in ways other than H3K9me3.

We also note that *set-32* mutants contained two types of nuclei. Some nuclei were indistinguishable from wild type (Fig 7, row 5) and some had reduced levels of H3K9me3 (Fig 7, row 6). The two *set-32* mutant nuclei depicted on Fig 7 come from the same worm, illustrating cell-to-cell variation within a single animal in this genetic background. These observations suggest that in contrast to what is seen in embryos [23], in differentiated cells, enzymes other than SET-25 contribute to the deposition of H3K9me3.

### The DCC remains X-bound and the X chromosomes maintain enrichment for H4K20me1

**Fig 8:**
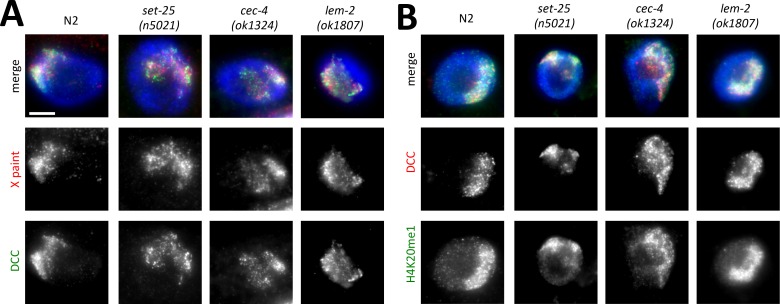
DCC localization and H4K20me1 enrichment in tethering mutants. **(A)** Combined X paint fluorescence in situ hybridization (red) and immunofluorescence with antibodies specific to DCC component DPY-27 (green). The DCC remains localized on the decondensed X chromosomes of tethering mutants. **(B)** Immunofluorescence images with antibodies specific to H4K20me1 (green) and DCC component CAPG-1 (red) to mark the location of the X chromosome. H4K20me1 remains enriched on DCC-bound X chromosomes. Scale bar, 5 μm.

A possible explanation for the dosage compensation defects in tethering mutants (Fig 1) is disruption of DCC localization. To test this possibility we stained worms with X-paint FISH probe followed by immunofluorescence using antibodies specific to the DCC subunit DPY-27. Despite changes in X chromosome morphology, we observed normal localization of the DCC to X chromosomes (Fig 8A). While we cannot exclude minor changes in DCC distribution along the X chromosome, we conclude that the DCC does associate with the X chromosomes in tethering mutants.

An alternative explanation for defects in dosage compensation in these mutants is that DCC function is disrupted. Previously characterized molecular functions of the DCC include condensation of the X chromosome [7, 39], altering chromosome topology [8], and leading to a different distribution of posttranslational histone modifications, particularly H4K20me1 and H4K16ac [9, 10]. To test whether mutations in tethering genes affect the ability of the DCC to lead to enrichment of H4K20me1 on the X, we co-stained worms with antibodies specific to the DCC (to mark the location of the X) and antibodies specific to H4K20me1. Results showed that this chromatin mark continues to be enriched on the X chromosomes (Fig 8B). Therefore, at least some aspects of DCC function remain intact in tethering mutants. Although wild type level of enrichment of H4K20me1 on the X appears to be required for X chromosome condensation [39], our results indicate that it is not sufficient.

### Derepression of X-linked genes in tethering mutants

**Fig 9:**
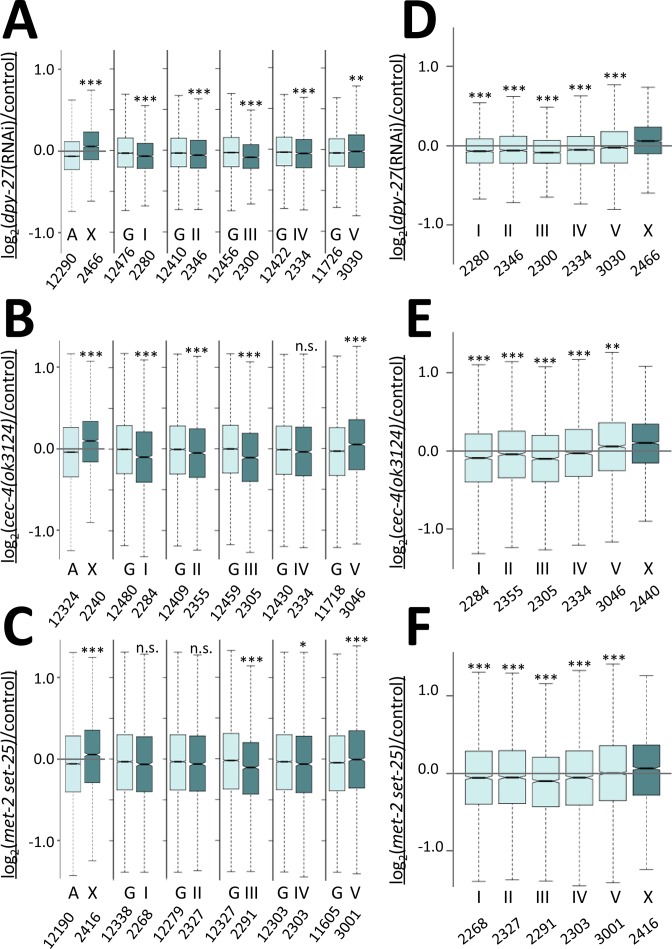
RNA-seq analysis of gene expression changes in tethering mutants. (A-C) Boxplots show the distribution of log_2_ expression ratios on the X chromosomes and autosomes (A), as well as all individual autosome (I, II, III, IV, V) and the rest of the genome (G) between *dpy-27(RNAi)* and control (A), *cec-4(ok3124)* mutant and control (B), and *met-2(n4256) set-25(n5021)* mutant and control (C). The X chromosome was significantly derepressed compared to autosomes, but the only autosome that showed derepression compared to the rest of the genome is chromosome V. (D-F) Boxplots show log_2_ expression ratios on the X chromosome and on individual autosomes in *dpy-27(RNAi)* (D), *cec-4(ok3124)* mutants (E), and *met-2(n4256) set-25(n5021)* mutants (F). The X chromosome is more derepressed than any individual autosome in all three backgrounds. Differences in gene expression changes from the X was tested between the X and all autosomes, or the X and individual autosomes by one-sided Wilcoxon rank-sum test, and between a given autosome and the rest of the genome by two-sided Wilcoxon rank-sum test (n.s. = not significant, * = p < 0.05, ** = p<0.01, *** = p < 0.001).

To test how loss of heterochromatic anchoring affects gene expression, we performed mRNA-seq analysis (Fig 9). We performed this analysis in L1 stage larval hermaphrodites. By this stage, somatic cells are differentiated, dosage compensation-mediated chromatin marks are fully established [9, 50], and gene expression differences resulting from DCC function are easily detectable using RNA-seq [11]. Based on the low level of male rescue observed upon RNAi-depletion of tethering genes (Fig 1), we did not expect major disruptions of regulation of X-linked genes. Therefore, to compare gene expression changes in tethering mutants to gene expression changes resulting from moderate changes in DCC function, we generated a data set using L1 hermaphrodite worms in which the DCC subunit DPY-27 was partially depleted by RNAi. Note that even though DPY-27 levels were significantly reduced in these worms (S4C Fig), there was very little lethality associated with the RNAi treatment, indicating that DCC function was only partially disrupted.

Under these mild *dpy-27(RNAi)* conditions, we observed a small increase in average X-linked gene expression compared to gene expression changes on autosomes. These results are qualitatively similar to previously reported analysis of dosage compensation mediated gene expression changes [8, 11, 12], but the magnitude of change is smaller, indicating that this data is an appropriate representation of gene expression changes when DCC function is partially disrupted. The median log_2_ ratio of expression between *dpy-27(RNAi)* worms and control vector RNAi treated worms was significantly higher on the X (0.062) compared to autosomes (-0.059) (for all expressed genes) (Fig 9A, one-sided Wilcoxon rank-sum test p = 3.09 x 10^−78^), consistent with a small degree of X depression. Strains carrying mutations in *cec-4(ok3124)* or *met-2(n4256) set-25(n5021)* showed similar X chromosome derepression compared to *dpy-27(RNAi)*. The median log_2_ ratio of expression between *cec-4(ok3124)*/control or *met-2(n4256) set-25(n5021)*/control was significantly higher on the X (0.095 and 0.057 respectively) compared to autosomes (-0.042 and -0.057 respectively) (Fig 9B and 9C, one sided Wilcoxon rank-sum test p = 8.07 x 10^−42^ and p = 8.48 x 10^−18^).

To examine whether the observed differences in gene expression might reflect random variations between chromosomes, we examined the average gene expression change on each autosome compared to the rest of the genome (Fig 9A–9C). Small gene expression change differences were in fact observed between any autosome and the rest of the genome, and many of these differences were statistically significant (two-sided Wilcoxon rank sum test). However, for chromosomes I, II, III, and IV, the autosome was downregulated, not upregulated, compared to the rest of the genome (Fig 9A–9C). Chromosome V was the only autosome that appeared upregulated compared to genomic average, both in *dpy-27(RNAi)* and in tethering mutants, and this derepression was mild compared the derepression observed for the X chromosome (Fig 9A–9C).

We then compared expression changes on the X chromosome to each autosome individually (Fig 9D–9F). Again we observed a small yet statistically greater level of derepression on the X than any of the autosomes in all three backgrounds. Importantly, the X chromosome was significantly more upregulated than chromosome V, the only autosome that is derepressed compared to the genomic average (Fig 9D–9F, one sided Wilcoxon rank-sum test p = 8.35 x 10^−24^ in *dpy-27(RNAi)*, p = 0.00126 in *cec-4*, and p = 0.000468 in *met-2 set-25*). These analyses indicate that the greatest degree of derepression is seen on the X chromosome. Furthermore, the trends were the same in *dpy-27(RNAi)* and in *cec-4* and *met-2 set-25* mutants, indicating again that gene expression changes in tethering mutants are comparable to gene expression changes in partial DCC depletion conditions. These results suggest that lack of CEC-4, or MET-2 and SET-25 function leads to similar gene expression changes as a partial depletion of the DCC.

To complement our analysis of average gene expression, we also looked at genes whose expression changed significantly using DESeq2 analysis (S5A Fig). Consistent with previous gene expression studies [22], expression of very few genes changed significantly in *cec-4* mutants. However, the same was true for *dpy-27(RNAi)*. In both cases, a slightly higher percentage of X-linked genes were upregulated than the percentage of upregulated autosomal genes, and a slightly higher percentage of X-linked genes were upregulated than downregulated. There were more genes with significant changes in gene expression in *met-2 set25* mutants, consistent with a dosage-compensation-independent gene regulatory role for these genes [22]. The percentage of X-linked genes that met the statistical criteria for significant upregulation was not greater than the percentage of downregulated X-linked genes in this background (S5A Fig). Perhaps the subtle changes caused by mild dosage compensation defects are not sufficient to show statistically significant changes in expression (based on 3 or 4 biological replicates) at the individual gene level.

**Fig 10:**
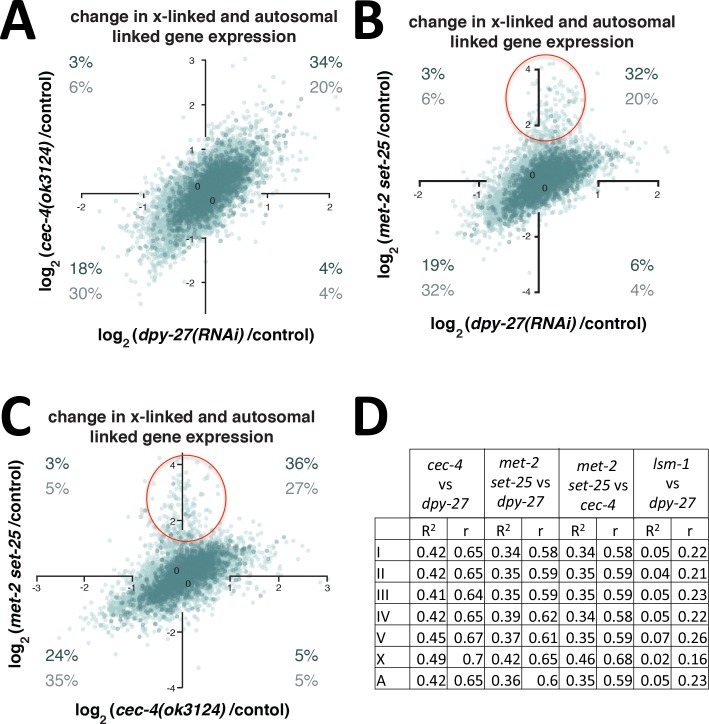
Comparison of gene expression changes in tethering mutants and partial DCC depletions. The magnitude of log_2_ expression ratios of X-linked (dark) and autosomal linked genes (light) between *cec-4(ok3124)* mutant and control plotted against *dpy-27(RNAi)* and control RNAi **(A)**, *met-2(n4256) set-25(n5021)* mutant and control plotted against *dpy-27(RNAi)* and control RNAi **(B)**, and *met-2(n4256) set-25(n5021)* mutant and control plotted against *cec-4(ok3124)* mutant and control **(C).** Red circles indicate a group of genes that are repressed by MET-2 and SET-25 independent of dosage compensation or *cec-4* function. Percent of X-linked (dark numbers) and autosomal genes (light numbers) with greater than 10% (log_2_ of 0.1) change in expression are indicated in each quadrant **(D)** The R-squared of each regression and the Pearson correlation values are shown for X-linked genes (X), for each individual autosome (I-V), and all autosomal genes (A) for each comparison. Values for a control analysis (*dpy-27* versus *lsm-1* mutants) are also indicated.

To further determine whether there is a correlation between the degree of gene expression change in the tethering mutants and the degree of gene expression change in worms with a partial defect in DCC function, we plotted the log_2_ ratio of expression of the tethering mutants and control worms against the log_2_ ratio of expression of *dpy-27(RNAi)* and control vector worms (Fig 10A and 10B). With a log_2_ cutoff of 0.1 (10%) for upregulation, the largest percentage of X-linked genes fell in the quadrant of derepression in both *dpy-27(RNAi)* and tethering mutants (32–34% versus 3–19% on other quadrants). For autosomal genes the opposite was true, and the largest percentage fell in the quadrant of downregulation in both backgrounds (30–32%, compared to 4–20% in other quadrants). These results indicate a bias toward upregulation of a common set of X-linked genes in DCC-deficient worms and in tethering mutants. A similar degree of correlation was observed when comparing *cec-4* to *met-2 set-25* (Fig 10C), and again the correlation was higher for X-linked genes than autosomal genes. There is a population of genes on autosomes whose expression is repressed by MET-2 and SET-25 independent of DCC-mediated changes (Fig 10B, red circle), or independent of CEC-4 (Fig 10C, red circle), consistent with a previous study [22].

To examine correlations between gene expression changes, we performed regression analysis, which showed a moderate positive correlation between tethering mutants and *dpy-27(RNAi)* log_2_ ratios for both X and autosomal genes (R-squared values ranged from 0.34 and 0.49, Pearson correlation values between 0.58 and 0.7) (Fig 10D). Additionally, X-linked genes had slightly higher R-squared and Pearson correlation values compared to autosomal genes. Correlations of gene expression changes on the X indicate that the genes whose expression is most affected by depletion of the DCC are also the genes whose expression is most affected in tethering mutants. Correlations on autosomes may be explained by the observation that defects in DCC activity affect not only X-linked gene expression, but indirectly also contribute to modulating autosomal gene expression [12]. A control analysis, gene expression changes in *dpy-27(RNAi)* correlated with gene expression changes in an unrelated condition (*lsm-1* mutants, [51]), showed lower R-squared values and lower Pearson correlation values on all chromosomes (Fig 10D).

To determine whether gene expression changes correlate with chromosomal changes, we compared log_2_ ratios of genes located at X chromosome left, middle, and right regions. Regions were designated based on LEM-2 ChIP-chip signals domains [26]. The region 0 Mb—4 Mb was designated "left", 4 Mb—15.75 Mb was designated "middle", and 15.75 Mb—17 Mb was designated "right". Since the middle region of the X chromosome is subject to the greatest level of decondensation and relocation in tethering and DCC mutants (Fig 4), we hypothesized that genes in the middle of the X would be more derepressed compared to the right and left arms. However, when examining the distribution of log_2_ ratios in *dpy-27(RNAi)/*control, *cec-4(ok3124)/*control, and *met-2(n4256) set-25(n5021)/*control, the X chromosome regions did not show significant differences by two-sided Wilcoxon rank-sum test (S5B Fig, median log_2_ ratio between 0.047 and 0.099 and p-values ranged from 0.09 and 0.90). While surprising, these observations are consistent with the model that DCC induced changes in X chromosome structure modulate gene expression chromosome-wide rather than locally [8, 11–13].

## Discussion

In a screen to identify genes with roles in X chromosome dosage compensation, we identified a group of genes with previously known roles in anchoring heterochromatic domains to the nuclear lamina: H3K9 HMTs, the chromodomain protein CEC-4, and the nuclear lamina protein LEM-2. These genes are collectively required to compact the X chromosomes and tether them to the nuclear periphery. Compartmentalization of the nucleus in this way may restrict availability of transcriptional activators for the X chromosomes, thus creating a repressive compartment to modulate X-linked gene expression. Although H3K9me and nuclear lamina interactions are enriched at the left end of the X chromosomes, we find that these mutations disproportionately affect compaction and subnuclear localization of the gene-rich middle portion. Large-scale changes in chromosome morphology are accompanied by only modest changes in gene expression, suggesting that while nuclear architecture does contribute to modulating gene expression, it is not the primary determinant.

### Models of the effect of heterochromatic anchors on X chromosome morphology

**Fig 11:**
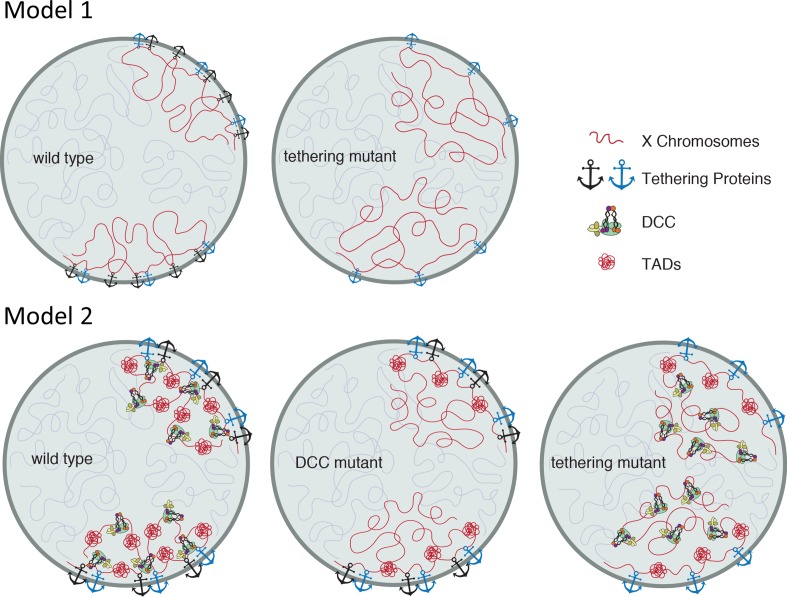
Model showing the effects of tethering and DCC function on X chromosome compaction and nuclear localization. Model 1: In differentiated cells, heterochromatin tethers (black anchors) and additional mechanisms (blue anchors) tether the left end of the X chromosome to the nuclear lamina. When heterochromatic tethers are lost, the left end of the X remains near the periphery, but the rest of the chromosome relocates to a more central position. Model 2: In wild type cells, the DCC organizes the X chromatin into topologically associating domains (TADs) and uses heterochromatic anchors to compact the X chromosome and bring it to the nuclear periphery. In the absence of the DCC, the left end of the X remains peripheral and compact due to the action of the tethering proteins, and its TAD structure is maintained. The rest of the X chromosome loses its TAD organization, decondenses and moves more internally. When heterochromatic anchors are lost, redundant tethers keep the left end of the X near the nuclear lamina, but the DCC is unable to compact the rest of the chromosome and bring it to the periphery.

The observation that the middle of the X chromosome is more sensitive to loss of heterochromatic anchors (Fig 4), can be explained by postulating the existence of redundant anchors, previously proposed to exist in differentiated cells [22, 23] (Fig 11, model 1). In this model, two types of anchors maintain peripheral localization of the X chromosome. Heterochromatic tethers are enriched at the left end of the X [23, 26], but the rest of the chromosome must also be weakly tethered. Additional anchors also tether the left end. When heterochromatic anchors are lost, the left end remains near the periphery due to the additional anchors, but the rest of the X chromosome decondenses and relocates centrally. However, if we assume that these tethers are not sex- and chromosome-specific, the model fails to explain why the X chromosome in males (Fig 5) and the autosomes in hermaphrodites (Fig 6) are not sensitive to the loss of heterochromatic tethers. To explain why only the DCC-bound X is affected, we propose an alternative model (Fig 11, model 2): (1) heterochromatic anchors at the left end of the X nucleate a compact chromatin structure, and (2) the activity of the DCC propagates this structural organization to encompass the entire chromosome. In the absence of the DCC, but in the presence of heterochromatic tethers (for example, the male X), the left end maintains its compact structure and peripheral localization. However, the rest of the chromosome decondenses and moves more centrally. In the presence of the DCC, but without heterochromatic anchors (tethering mutants), redundant anchors keep the left end at the periphery, but the DCC is unable to compact the rest of the chromosome and bring it to the periphery. Since autosomes are not bound by the DCC, they are not affected by the loss of heterochromatic tethers.

### Correlation between compaction, subnuclear localization and gene expression

Chromatin compaction and subnuclear localization are believed to be coordinated with gene expression levels to a certain degree [14–16]. We analyzed chromatin condensation, subnuclear localization, and gene expression changes in DCC-depleted animals and in tethering mutants. We observed elevated levels of X-linked gene expression (Fig 9), decreased compaction (Figs 2 and 4), and relocation to a more central position (Figs 3, 4 and 5), both in the absence of the DCC and in tethering mutants, providing support for the hypothesis that these processes are coordinated. However, the correlation is not perfect. The degree of gene expression change did not correlate well with the degree of decondensation and/or subnuclear relocation. At the whole chromosome level, the X chromosomes in tethering mutants decondensed to a degree comparable to DCC mutants (Fig 2). Similarly, the degree of relocation was greater in tethering mutants than in partial loss-of-function DCC mutant, and comparable to the positioning in XO animals that completely lack DCC function (Figs 3 and 5). However, gene expression changes in tethering mutants are much less significant than in DCC mutants (Fig 9). Similar conclusions were reached when we analyzed different regions of the X chromosome: relocation and decondensation was most significant in the middle of the X chromosome (Fig 4), but gene expression changes were comparable in all regions of the chromosome (S5 Fig). A higher resolution study may reveal a stronger correlation, but at the level of whole chromosomes, or large chromosomal domains, the correlation between gene expression change, chromosome decondensation and subnuclear localization is limited. A recent study showed that chromatin decondensation, even in the absence of transcriptional activation, is sufficient to drive nuclear reorganization [52]. Similarly, in *cec-4* mutants, decondensation of transgenic arrays is coupled to their relocation within the nucleus, but it is accompanied by only minimal changes in gene expression [22]. This is reminiscent of our results, where chromatin decondensation and relocation in general correlate, but the degree of condensation does not reflect the degree of gene expression change.

We believe these results reflect that fact that repression by the DCC involves multiple mechanisms, and disruption of condensation and subnuclear localization is not sufficient to cause major changes in gene expression. Other DCC-mediated changes, for example enrichment of H4K20me1 on the X chromosome, are intact in tethering mutants (Fig 8), and are sufficient to maintain repression. However, it should be emphasized that loss of tethers (and/or the accompanying change in X chromosome packaging and nuclear organization) does result in gene expression changes that are biologically significant. While the gene expression change is modest (Figs 9 and 10), it is sufficient to rescue a significant proportion of males in our genetic assay (Figs 1 and S1). Thus, chromatin condensation, subnuclear localization, and tethering to the nuclear periphery, may not be the primary determinants of gene expression change, or may act redundantly with other factors, but they do contribute to stabilizing gene expression programs in development [22] and during dosage compensation (this study).

### The role of condensin in interphase nuclear organization

Condensin has been implicated previously in chromosome territory organization in a variety of organisms [39, 53, 54]. In condensin mutant fission yeast, disruption of condensin-dependent intrachromosomal interactions disturbed chromosome territory organization [55]. We previously showed that the dosage compensation condensin complex is required for compaction of the X chromosomes in interphase in *C*. *elegans* [39]. Our current data reveal that interphase chromosome compaction requires not only the DCC, but also nuclear lamina anchors. We favor the interpretation that heterochromatic tethers and the DCC cooperate to compact the X chromosomes (Fig 11, model 2). Although low resolution, our FISH analysis supports this hypothesis. The X chromosomes appear compact and in a well-defined peripheral territory only when tethered and DCC-bound. In both DCC and tethering mutants, the X paint signal becomes more diffuse with less well-defined borders (Fig 2, [39]). Chromosome I paint signals in wild type worms qualitatively are more comparable to X paint signals in DCC mutants or tethering mutants than to X paint signals in wild type (Fig 6). The two ends of chromosome I are anchored to the nuclear periphery, and remain anchored in tethering mutants, while the middle domain is more centrally located even in wild type worms, reminiscent of the organization of X chromosome in tethering mutants and in males (Fig 6, [39]). Overall, these observations suggest that the DCC and heterochromatic anchors work together to compact and peripherally relocate the middle domain of the X chromosomes not directly tethered to the nuclear periphery.

It is interesting to note that we observe significant chromosome decondensation despite normal DCC binding to the chromosome (Fig 8). Current models of DCC binding to the X include a recruitment step to *rex* sites [12, 56, 57], which have very high levels of DCC binding [12, 58], and tend to define TAD boundaries [8]. From these *rex* sites to DCC spreads to *dox* sites enriched at promoter regions [12, 58]. From our low-resolution immunofluorescence analysis, DCC binding seems unaffected in tethering mutants. Yet, despite near normal levels of DCC, the X chromosome is not compacted, indicating that in the absence of heterochromatic tethers, DCC function appears to be compromised.

Our results also reveal parallels with recent genome-wide chromosome conformation capture (Hi-C) analysis of dosage compensated X chromosomes [8]. Hermaphrodite X chromosomes are packaged into a structure with regularly spaced boundaries between topologically associated domains (TADs). In the absence of the DCC, boundaries become less well defined and TAD organization weakens, except at the left end of the X, which is the domain that is tethered to the nuclear lamina [8] (Fig 11, model 2). This parallels our observations that in DCC mutants the left end of the X chromosome remains less affected than the rest of the chromosome. It is likely that the observed changes in TAD formation [8] and chromosome compaction and subnuclear localization (Figs 2–5, [39]) in the absence of the condensin-like DCC reflect the same underlying changes in chromosome structure analyzed at different resolutions and using different methods.

If TAD formation, chromatin condensation, and subnuclear localization indeed correlate, our results would predict that TADs at the left of the X chromosome would also be less disrupted in tethering mutants than along the rest of the X chromosome. These results and predictions would suggest that nuclear lamina anchors (both the anchors mediated by H3K9me3 and the yet uncharacterized anchors) are able to impose this level of organization (TAD formation) on the tethered portion of the chromosome. Autosomes in general lack regularly spaced TADs, except at the tethered ends of chromosome arms [8]. This observation is consistent with our observations of peripherally located chromosome I arms (Fig 6), and our suggestion that nuclear lamina anchors are sufficient to form regularly spaced TADs in the anchored domain.

### Changes in nuclear organization during cellular differentiation and development

The mechanisms of anchoring appear to be different in embryonic cells compared to differentiated cells. Repetitive heterochromatic arrays require H3K9 methylation and CEC-4 for peripheral localization in embryonic cells but not in differentiated cells, suggesting that differentiated cells have other mechanisms in place for tethering genomic regions to the nuclear envelope [22, 23]. Whether heterochromatin and CEC-4 mediated anchors continue to function in differentiated cells remained unclear [22], yet our results are consistent with this possibility. The left end of the X chromosome remains in the vicinity of the nuclear lamina in the absence of H3K9me3, LEM-2, or CEC-4 in fully differentiated cells, suggesting the existence of additional anchors (Fig 4). However, X chromosome morphology does change in the absence of these proteins, indicating that tethers mediated by them continue to influence chromosome structure in differentiated cells.

Our results are reminiscent of the findings in differentiating mouse cells [59]. In early development, lamin B receptor (*Lbr*) is the predominant mediator of interactions with the nuclear lamina. Later in development, lamin-A/C-dependent tethers appear, sometimes accompanied by the loss of *Lbr*-mediated mechanisms. Loss of peripheral localization of heterochromatin is only observed when both types of tethers are absent [59]. It will be interesting to uncover the nature of the additional anchors in differentiated *C*. *elegans* tissues and how these anchors affect X chromosome morphology and dosage compensation. However, it is possible that the additional anchors will be cell-type specific, consistent with the observation in mammalian cells where various tissue-specific transmembrane proteins are used to anchor genomic regions to the nuclear lamina [60]. Tissue-specific differences between anchoring mechanisms are also consistent with observations that point mutations in lamin can exhibit tissue-specific defects in humans [61] as in *C*. *elegans* [21].

### Summary

In a screen for genes that promote dosage compensation in *C*. *elegans*, we identified a group of genes implicated in anchoring heterochromatin to the nuclear lamina. When these genes are not functional, the X chromosome decondenses and moves away from the nuclear periphery. Decondensation and subnuclear relocation mostly affects the gene-rich middle portion of the X chromosome, while the tethered left end is less affected. We propose that the DCC uses these heterochromatic anchors to condense and position the X chromosome near the nuclear periphery. Moving the X chromosome into this peripheral compartment contributes to lowering X-linked gene expression levels. Establishment of this nuclear compartment as a way to regulate the X chromosome is consistent with previous observations [8, 12, 13] and our results (this study), which found no correlation between DCC binding, DCC induced chromosomal changes, and repression of gene expression.

## Materials and Methods

### *C*. *elegans* strains

Strains were maintained as described [62]. Strains include: N2 Bristol strain (wild type); MT16973 *met-1(n4337)* I; VC967 *set-32(ok1457)* I; VC1317 *lem-2(ok1807)* II; MT13293 *met-2(n4256)* III; PFR40 *hpl-2(tm1489)* III; MT17463 *set-25(n5021)* III; EKM104 *set-25(n5021)* III; *him-8(mn253)* IV; EKM99 *met-2(n4256) set-25(n5021)* III; RB2301 *cec-4(ok3124)* IV; TY4403 *him-8(e1489)* IV; *xol-1(y9) sex-1(y263)* X; TY1072 *her-1(e1520)* V; *sdc-2(y74*) X; EKM71 *dpy-21(e428)* V; RB1640 *set-20(ok2022)* X; VC2683 *set-6(ok2195)* X; PFR60 *hpl-1(tm1624)* X. Males were obtained from strains that carry a mutation in *him-8*, a gene required for the segregation of the X chromosome in meiosis, mutations in which lead to high incidence of males, but do not affect the soma. All strains were fed OP50 and grown at 15°C to avoid temperature sensitive sterility associated with some mutations in some the strains.

### RNA Interference (RNAi)

*E*. *coli* HT115 bacteria cells carrying plasmids that express double stranded RNAi corresponding to the gene of interest, were grown from a single colony for 8–10 hours at 37°C and 125 μL were plated onto NGM plates supplemented with IPTG (0.2% w/v) and Ampicillin (1ug/ml) and allowed to dry overnight. For imaging experiments, worms were grown on RNAi plates for two generations at 15°C as follows: L1 worms were placed on a plate and allowed to feed until they reached L4 stage whereby 2–3 L4 worms were moved to a new plate and allowed to lay eggs for 24 hours. F1 worms were grown to 24 hours post L4 for fixation. The male rescue RNAi screen was described in detail in [35]. Briefly, *him-8(e1489)IV; xol-1(y9) sex-1(y263) X* worms were treated with RNAi as before. For results shown on Fig 1, L4 worms from the P0 generation were allowed to lay eggs for 24hr at 20°C, the parents were removed, and embryos were counted. For results shown on S1 Fig, P0 worms were fed RNAi food for an additional day, until they reached young adult stage before egg collection began. Worms were grown at 20°C and males were counted and removed for 2–4 days after eggs were laid. Male rescue was calculated by dividing the number of observed males by the number of expected males. The *him-8(e1489)IV* strain consistently yields 38% male progeny so the expected number of males was assumed to be 38% of the embryos laid. Male rescue was calculated as: (Observed number of males)/ (0.38 x number of eggs laid).

### Antibodies

The following antibodies were used: rabbit anti-H3K9me3 (Active Motif #39766), rabbit anti-H4K20me1 (Abcam ab9051), rabbit anti-DPY-27 [4], rabbit anti-beta tubulin (Novus NB600-936). Anti-CAPG-1 antibodies were raised in goat using the same epitope as in [4]. Secondary anti-rabbit and anti-goat antibodies were purchased from Jackson Immunoresearch.

### Immunofluorescence

Immunofluorescence experiments were performed as described [4]. Young adult worms were dissected in 1X sperm salts (50 mM Pipes pH 7, 25 mM KCl, 1 mM MgSO4, 45 mM NaCl and 2 mM CaCl2, supplemented with 1 mM levamisole), fixed in 2% paraformaldehyde in 1X sperm salts for 5 minutes and frozen on dry-ice for 10 minutes. For anti-H4K20me1 and anti-CAPG-1 staining, worms were fixed in 1% PFA. After fixation, slides were frozen on a dry ice block for 20–30 minutes, washed three times in PBS with 0.1% Triton X-100 (PBST) before incubation with diluted primary antibodies in a humid chamber, overnight at room temperature. Slides were then washed three times with PBST, incubated for 4 hours with diluted secondary antibody at room temperature, washed again twice for 10 minutes each with PBST, and once for 10 minutes with PBST plus DAPI. Slides were mounted with Vectashield (Vector Labs). Antibodies were used at the following concentrations: CAPG-1, 1:1000; DPY-27, 1:100; H4K20me1, 1:200; H3K9me3, 1:500.

### Fluorescence In Situ Hybridization (FISH)

Slides were prepared as for immunofluorescence through the PBST washes following fixation. Slides were then dehydrated with sequential 2 minute washes in 70%, 80%, 95% and 100% ethanol before being allowed to air dry for 5 minutes at room temperature. Full X-paint probe and chromosome I paint probe preparation was described in detail in [39, 63]. The X-left probe contained DNA amplified from the following YACs: Y35H6, Y47C4, Y51E2, Y02A12, Y105G12, Y97B8, Y76F7, Y40,H5, Y43D5, Y18F11, Y89H11 (covers the region from 0.1Mb to 4.2 Mb of the chromosome). The X-mid probe contained DNA amplified from the following YACs: Y18C11, Y50C2, Y70G9, Y44D2, Y102D2, Y97D4, Y97D9 (covers the region from 7.4Mb to 11.0 Mb). The X-right probe contained DNA amplified from the following YACs: Y31A8, Y52C11, Y42D5, Y53A6, Y7A5, Y46E1, Y50B3, Y25B5, Y43F3, Y52F1, Y68A3 (covers the region from 14.0 Mb to 17.6 Mb of the chromosome). The Chromosome I left probe was made from the following YACs: Y73F10, Y50C1, Y65B4, Y18H1, Y73A3, Y34D9, Y48G8, Y52D1, Y71G12, Y102E12, Y71F9, Y115A10, Y44E3, Y74A12, Y74A11, Y39E12, Y40G6, Y110A7 (covers the region from the 0.2–4.6 Mb of the chromosome); chromosome I middle probe was made from the following YACs: Y70C6, Y46D1, Y54B12, Y101C10, Y39A9, Y53F1, Y97F9, Y97D1, Y97E2, Y43C3, Y43E2, Y49G9, Y102E5, Y106G6 (covering the region from 4.6 Mb—10.1 Mb); the chromosome I right probe was made form the following YACs: Y71B8, Y19G12, Y37F4, Y95D11, Y53A2, Y47H9, Y47H10, Y45E10, Y91F4, Y50A7, Y43D10, Y40B1, Y63D3, Y112D2, Y54E5 (covering the region fro 10.1–15.07 Mb). 10 microliters of probe was added to each slide, covered with a coverslip and placed on a 95°C heat block for 5 minutes. The heat block was then cooled to 37°C slowly and the slides were moved to a 37°C incubator in a humid chamber and incubated overnight. Slides were washed as follows: 3 washes of 2X SSC/50% formamide for 5 minutes each; 3 washes of 2X SSC for 5 minutes each; 1 wash of 1X SSC for 10 minutes. All washes were performed in a 39°C water bath. Finally, the slides were washed once with PBST containing DAPI for 10 minutes at room temperature before mounting with Vectashield.

### Quantification

Volume Quantification: Chromosome volumes were quantified as in [39]. Briefly, using the Mask: Segment function of Slidebook, a user-defined threshold is determined that separates signal from background and auto-fluorescence. The same level of background was used for all nuclei based on observed background. Masks were calculated for each channel with DAPI being the primary mask and the X paint being the secondary mask. Nuclear volume was calculated by taking the number of voxels (volumetric pixels) for the DAPI channel to determine total DNA content (morphology: volume (voxels)). The overlapping voxels between the X and the DAPI was determined by using a cross mask of the DAPI and X paint signals (cross mask: mask overlaps) in Slidebook. The percent nuclear volume occupied by the X was determined by dividing the number of X voxels by the total number of DAPI voxels.

Three-zone assay quantification: Concentric ovals of equal area were drawn over one focal plane from the center of the Z stack that contained the largest amount of X FISH signal. Masks were made from each of these zones using the Advanced operations > Convert regions to mask objects function in Slidebook. A single plane from the X chromosome mask set for volume quantification was used here. The amount of X signal in each of the zones was calculated using the cross mask: mask overlap function in Slidebook where the zone mask was the primary mask and the X mask was the secondary mask. The total voxels for all three zones were summed and the voxels in each zone were divided by the total to determine what percentage of the X signal was located in each zone.

### mRNA-seq

Worms were synchronized by bleaching gravid adults to isolate embryos and allowing worms to hatch overnight. Newly hatched L1 larval worms were plated and grown for 3 hours on NGM plates with OP50. To the worm pellet, ten volumes of Trizol were added and RNA was extracted and precipitated using the manufacturer's instructions. Total RNA was cleaned using the Qiagen RNeasy kit. Non-stranded mRNA-seq libraries were prepared using TruSeq RNA Library Preparation Kit. Single-end 50-bp sequencing was performed using Illumina HiSeq-2000. Reads were trimmed for quality using the TrimGalore program from Babraham Bioinformatics (http://www.bioinformatics.babraham.ac.uk/projects/trim_galore/) and aligned to the *C*. *elegans* genome version WS235 with Tophat v 2.0.13 (Trapnell et. al. 2012). Default parameters allow up to 20 hits for each read. Gene expression was quantified using Cufflinks v2.2.1 with use of “rescue method” for multi-reads and supplying gene annotation for WS235. Gene count estimation was performed using HTSeq-count tool v0.6.0 in the default “union” mode (Anders et. al. 2014). Differential expression analysis was performed using DESeq2 v1.6.3 in R version 3.2.3 (Anders and Huber 2010; R Development Core Team 2012). All analyses were performed with genes that had average expression level above 1 RPKM (fragments per kilobase per million, as calculated by Cufflinks).

### Western blot

From the worm suspension collected for RNA-seq experiments, 50 μL of L1s were used for protein analysis. For CAPG-1 antibody validation, 50 μL of mixed stage worms were used. Equal volume of sample buffer was added (0.1 M Tris pH 6.8, 7.5 M urea, 2% SDS, 100mM β-ME, 0.05% bromophenol blue), the suspension was heated to 65°C for 10 minutes, sonicated for 30-seconds twice, heated to 65°C for 5 minutes, 95°C for 5 minutes, then kept at 37°C until loading onto SDS-PAGE gel. Proteins were transferred to nitrocellulose and probed with the appropriate antibodies.

## Supporting Information

S1 Fig Additional male rescue analysis. A limited number of genes were analyzed in each experiment (**A, B,** and **C**), but using four independent biological replicates. Note that RNAi feeding of parents was extended by 24 hours compared to the experiment shown on Fig 1. This led to higher levels of male rescue overall, but the trend remained the same. OP50 is the normal bacterial food source, without any plasmid to produce RNA. With the exception of *set-6* and *set-20*, RNAi of all genes rescued significantly more males than control vector RNAi. It is important to point out that the few males rescued on vector RNAi plates were small and sickly, while the males rescued using RNAi of the other genes appeared more normal size and had better mobility. Error bars indicate standard deviation based on four replicates. Asterisks indicate statistical significance using Student t-test, n.s. = p>0.5, * = p<0.05, ** = p<0.01, *** = p<0.001. Numbers of embryos counted and p-values (compared to vector RNAi) are shown in the table below each graph.
(TIF)

S2 Fig Chromosome volume measurements in hypodermal nuclei of hermaphrodites. **(A)** X chromosome paint FISH (red) in diploid tail tip hypodermal nuclei (DAPI, blue) of hermaphrodite adult worms. The X chromosomes are compact and peripherally localized in wild type (N2), but are decondensed and more centrally located in mutants. Scale bar, 1 μm. **(B)** Quantification of X chromosome volumes normalized to nuclear size (n = 17–26 nuclei). Error bars indicate standard deviation. *** = p<0.001 by Student's t-test (N2 compared to appropriate mutant).
(TIF)

S3 Fig X paint FISH images in irregularly-shaped nuclei. **(A)** Representative irregularly shaped nuclei in the various backgrounds. The X is compact and peripherally located in N2 hermaphrodites and is decondensed and more centrally located in tethering mutants and in males. **(B)** Table indicating the percent of nuclei in each background that were suitable for analysis using the three-zone assay.
(TIF)

S4 Fig Antibody validation and RNAi-depletion control. **(A)** Immunofluorescence analysis of the newly developed CAPG-1 antibody in nuclei of control vector RNAi-treated worms shows two territories corresponding to the X chromosomes. In *capg-1(RNAi)* nuclei, the signal is below level of detection, similar to what has been observed previously with other antibodies to DCC components. (B) On a western blot, the antibody recognizes a protein of the predicted size (131 kD) in control vector RNAi treated worms, but not in CAPG-1 RNAi treated worms. Tubulin was used as loading control. **(C)** Western blot analysis of three control and three *dpy-27(RNAi)* samples, indicating levels of DPY-27 depletion. Tubulin is shown as a loading control.
(TIF)

S5 Fig Additional analysis of gene expression changes. (A) Numbers and percentages of genes with significantly changed levels of gene expression (DESeq2, padj<0.1 and padj<0.05) on the X chromosome and the autosomes in each background. (B) Boxplots show the distribution of log_2_ expression ratios on X chromosome regions between *dpy-27* and control RNAi, *cec-4(ok3124)* mutant and control, and *met-2(n4256) set-25(n5021)* mutant and control. Expression differences between X regions were tested by two-sided Wilcoxon rank-sum test. No significant differences were found.
(TIF)

S1 File **List of mRNA-seq data sets and their GEO accession numbers (tab 1), average RPKM expression levels for mRNA-seq data sets (tab 2), and DEseq2 analysis results for differential expression (tab 3)**.
(XLSX)

S1 Table Statistical analysis of X chromosome FISH data using the three-zone assay. (PDF)

S2 Table Statistical analysis of chromosome I FISH data using the three-zone assay. (PDF)
