# Supplementary figures and images for: Anchoring of Heterochromatin to the Nuclear Lamina Reinforces Dosage Compensation-Mediated Gene Repression
Source: PLoS Genet. 2016 Sep 30;12(9):e1006341. doi: 10.1371/journal.pgen.1006341 (PMC5045178; doi:10.1371/journal.pgen.1006341)

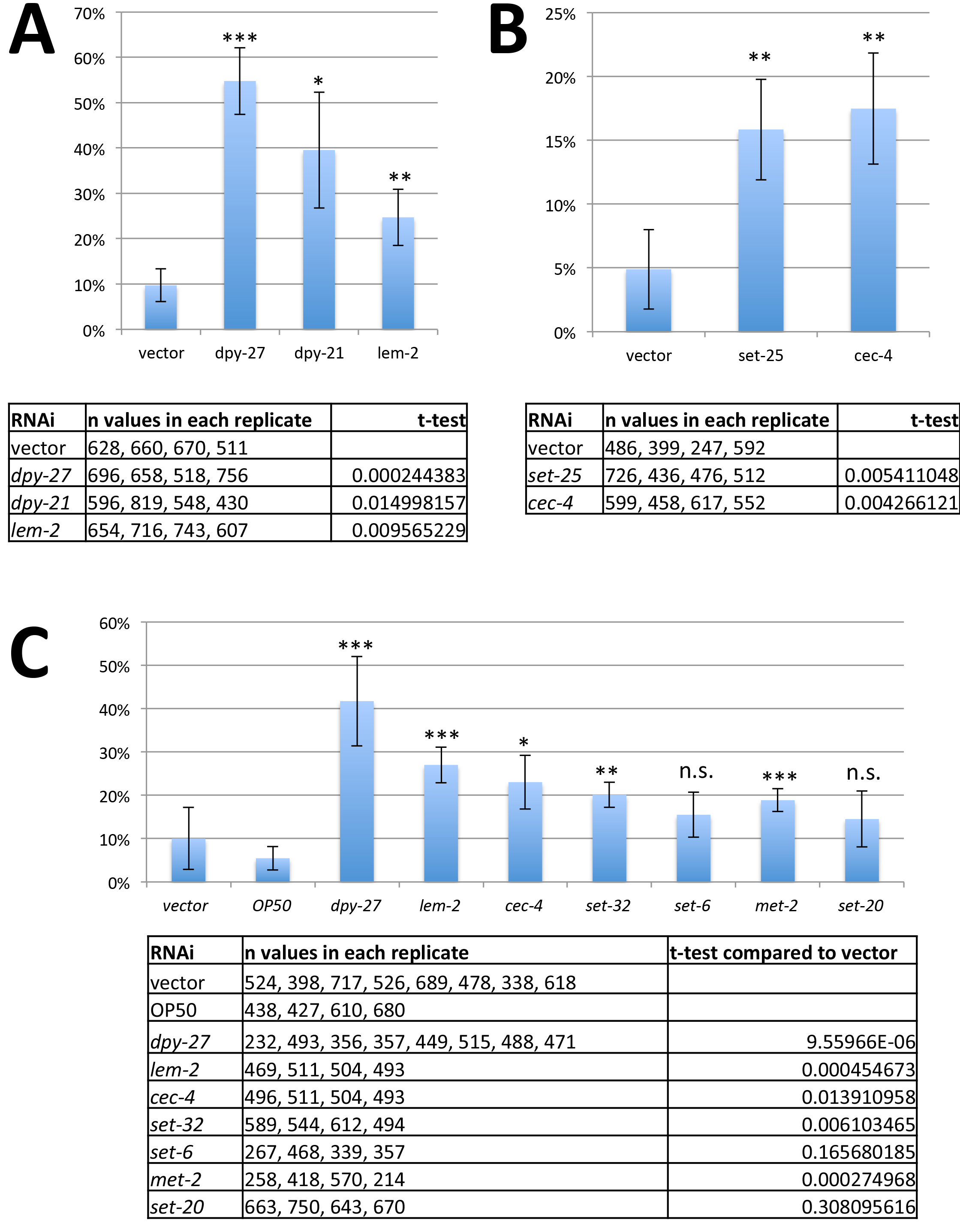

Supplement: S1 Fig — A limited number of genes were analyzed in each experiment (A, B, and C), but using four independent biological replicates. Note that RNAi feeding of parents was extended by 24 hours compared to the experiment shown on Fig 1. This led to higher levels of male rescue overall, but the trend remained the same. OP50 is the normal bacterial food source, without any plasmid to produce RNA. With the exception of set-6 and set-20, RNAi of all genes rescued significantly more males than control vector RNAi. It is important to point out that the few males rescued on vector RNAi plates were small and sickly, while the males rescued using RNAi of the other genes appeared more normal size and had better mobility. Error bars indicate standard deviation based on four replicates. Asterisks indicate statistical significance using Student t-test, n.s. = p>0.5, * = p<0.05, ** = p<0.01, *** = p<0.001. Numbers of embryos counted and p-values (compared to vector RNAi) are shown in the table below each graph. (TIF) [file pgen.1006341.s001.tif]

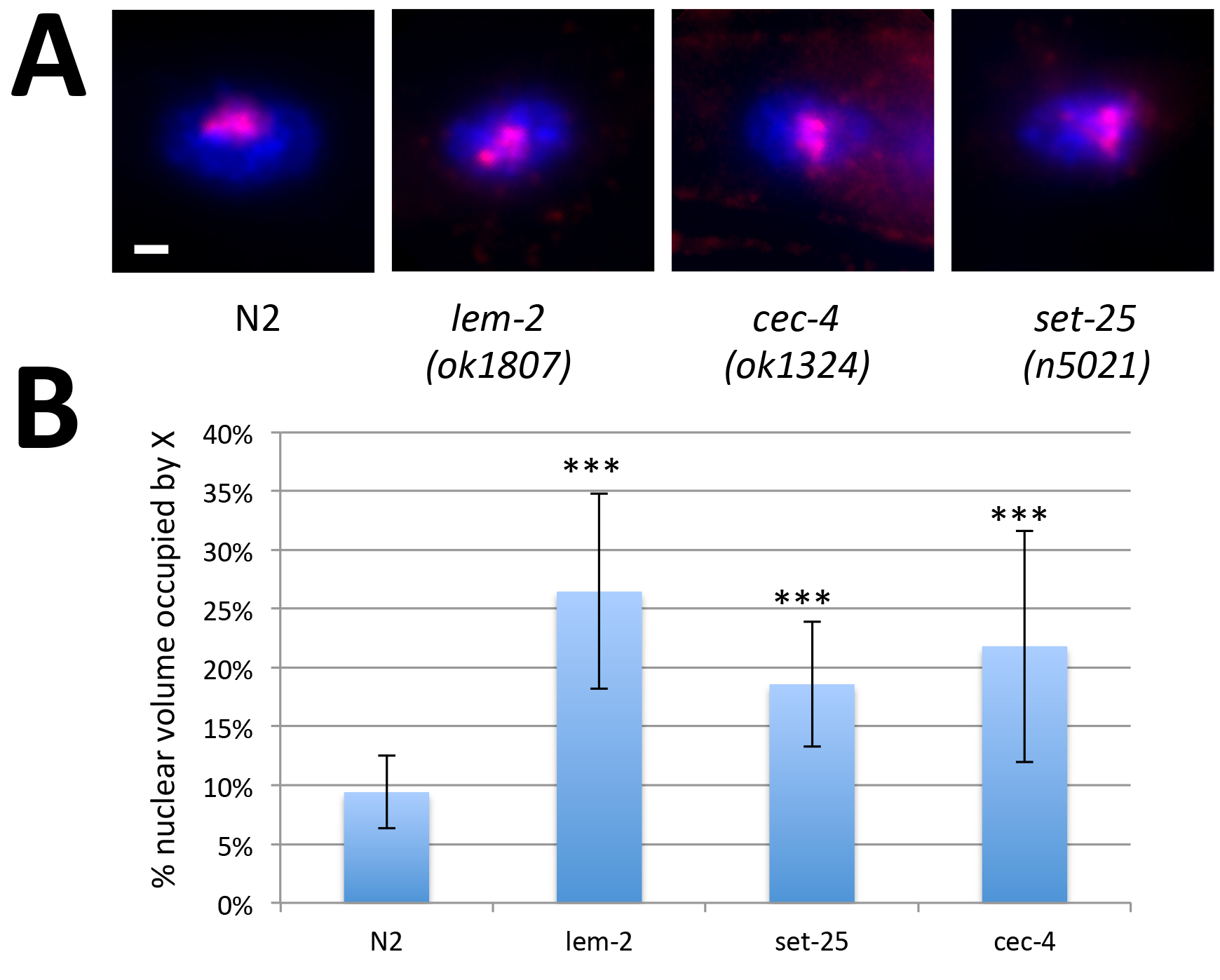

Supplement: S2 Fig — (A) X chromosome paint FISH (red) in diploid tail tip hypodermal nuclei (DAPI, blue) of hermaphrodite adult worms. The X chromosomes are compact and peripherally localized in wild type (N2), but are decondensed and more centrally located in mutants. Scale bar, 1 μm. (B) Quantification of X chromosome volumes normalized to nuclear size (n = 17–26 nuclei). Error bars indicate standard deviation. *** = p<0.001 by Student's t-test (N2 compared to appropriate mutant). (TIF) [file pgen.1006341.s002.tif]

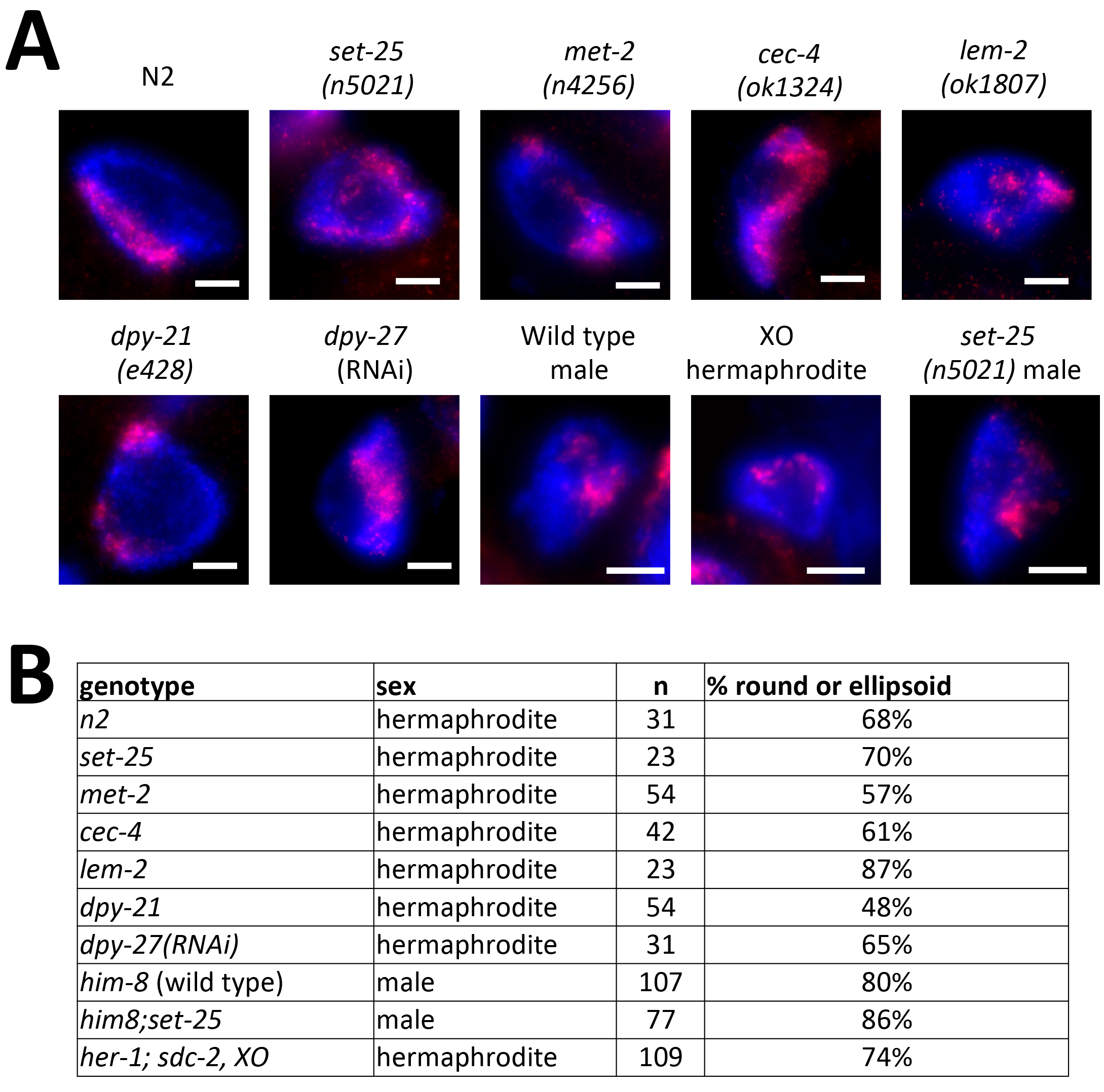

Supplement: S3 Fig — (A) Representative irregularly shaped nuclei in the various backgrounds. The X is compact and peripherally located in N2 hermaphrodites and is decondensed and more centrally located in tethering mutants and in males. (B) Table indicating the percent of nuclei in each background that were suitable for analysis using the three-zone assay. (TIF) [file pgen.1006341.s003.tif]

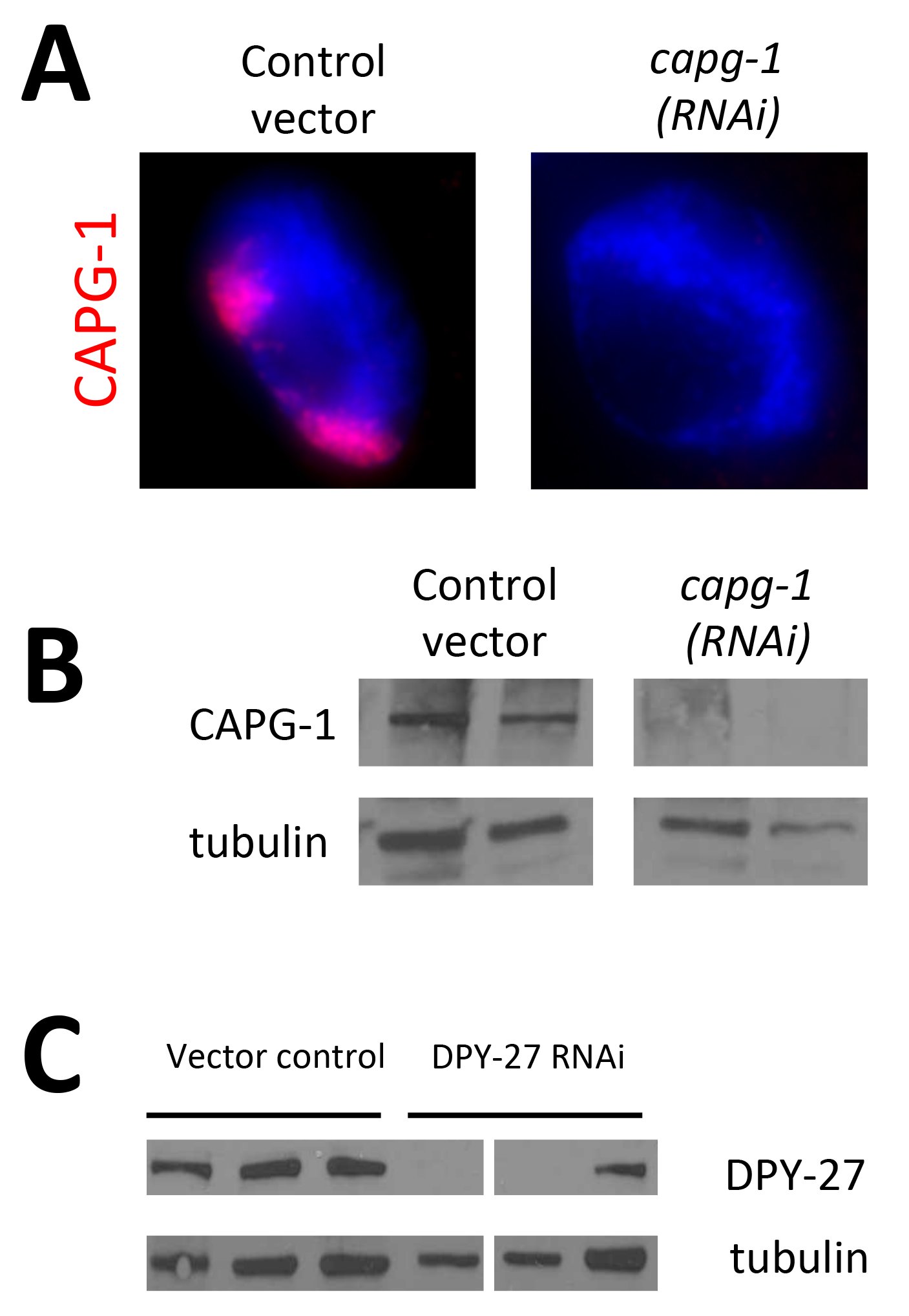

Supplement: S4 Fig — (A) Immunofluorescence analysis of the newly developed CAPG-1 antibody in nuclei of control vector RNAi-treated worms shows two territories corresponding to the X chromosomes. In capg-1(RNAi) nuclei, the signal is below level of detection, similar to what has been observed previously with other antibodies to DCC components. (B) On a western blot, the antibody recognizes a protein of the predicted size (131 kD) in control vector RNAi treated worms, but not in CAPG-1 RNAi treated worms. Tubulin was used as loading control. (C) Western blot analysis of three control and three dpy-27(RNAi) samples, indicating levels of DPY-27 depletion. Tubulin is shown as a loading control. (TIF) [file pgen.1006341.s004.tif]

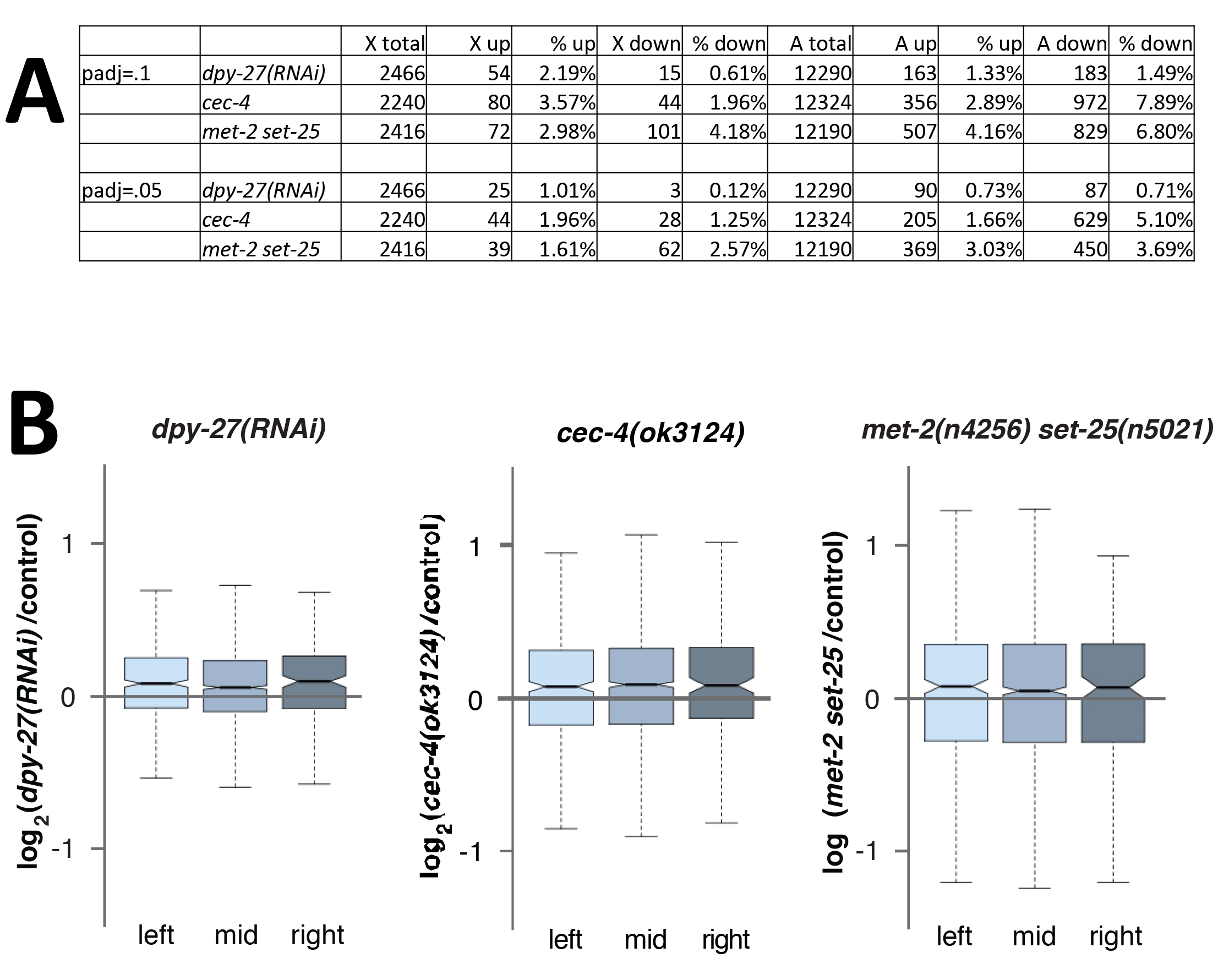

Supplement: S5 Fig — (A) Numbers and percentages of genes with significantly changed levels of gene expression (DESeq2, padj<0.1 and padj<0.05) on the X chromosome and the autosomes in each background. (B) Boxplots show the distribution of log2 expression ratios on X chromosome regions between dpy-27 and control RNAi, cec-4(ok3124) mutant and control, and met-2(n4256) set-25(n5021) mutant and control. Expression differences between X regions were tested by two-sided Wilcoxon rank-sum test. No significant differences were found. (TIF) [file pgen.1006341.s005.tif]
