# Supplementary material for: Anchoring of Heterochromatin to the Nuclear Lamina Reinforces Dosage Compensation-Mediated Gene Repression
Source: PLoS Genet. 2016 Sep 30;12(9):e1006341. doi: 10.1371/journal.pgen.1006341 (PMC5045178; doi:10.1371/journal.pgen.1006341)
Supplement: S1 Table — (PDF) [file pgen.1006341.s007.pdf]

whole X paint

	N2	*set-25*	*met-2 set-25*	*cec-4*	*lem-2*	*dpy-21*	*dpy-27(RNAi*	XO herm	wt male	set-25 male
n	10	10	10	10	10	10	10	10	10	10
peripheral %	0.2460166	0.18108734	0.17100431	0.12483749	0.14885454	0.23171619	0.15055586	0.14006767	0.10578356	0.08473489
st dev	0.12317077	0.08394753	0.09806461	0.07869138	0.08338365	0.17584326	0.10072818	0.06415232	0.07645738	0.07217885
intermed %	0.51555485	0.38185777	0.37545447	0.33605121	0.33670623	0.42227491	0.42842662	0.49212446	0.36320889	0.26202141
st dev	0.11855283	0.05666637	0.08394526	0.08337165	0.06486988	0.12696711	0.09175781	0.09170447	0.08244027	0.12066364
central %	0.23842855	0.43705489	0.45354122	0.5391113	0.51443923	0.3460089	0.42101753	0.36780787	0.53100755	0.6532437
st dev	0.09564744	0.10651531	0.13278119	0.11776565	0.11001907	0.22261633	0.1343343	0.126949	0.13926637	0.17946691
t-test of central ring compared to N2 hermaphrodite										
		0.00085208	0.00070257	9.5192E-06	2.3491E-05	0.18809096	0.00320924	0.02177761	5.6208E-05	3.7175E-06
t-test of central ring compared to set-25 hermaphrodite								t-test compared to wt male		
			0.76784616							0.09960997

left X probe

	N2	set-25	lem-2	cec-4	dpy-27(RNAi)
n	12	12	12	12	12
peripheral %	0.24059006	0.19880636	0.08421264	0.16131613	0.16444175
st dev	0.12888683	0.16120025	0.08460705	0.13741298	0.17310133
intermed %	0.60562542	0.50227208	0.6161937	0.53208363	0.54276048
st dev	0.06694305	0.12348118	0.15840007	0.18913377	0.18914569
central %	0.15378454	0.29892157	0.29959368	0.30660023	0.29279777
st dev	0.13005938	0.14996516	0.20293387	0.25018588	0.24752464
t-test of central ring compared to N2 hermaphrodite					
		0.01895785	0.04995183	0.07821709	0.10355896

Middle X probe

	N2	set-25	lem-2	cec-4	dpy-27(RNAi	XO herm	wt male	set-25 male
n	12	12	12	12	12	10	10	10
peripheral %	0.219341	0.13426402	0.18524328	0.23265349	0.31545998	0.17023531	0.12021431	0.09025599
st dev	0.17906491	0.10996228	0.12817088	0.08833383	0.2030099	0.12669977	0.07361085	0.08042172
intermed %	0.61054607	0.35300007	0.37657471	0.39334755	0.41918957	0.40027687	0.28490383	0.30690104
st dev	0.16494192	0.08933676	0.14280516	0.08937391	0.09451049	0.18146025	0.17001368	0.16435189
central %	0.17011293	0.51273591	0.43818202	0.37399896	0.26535045	0.42948782	0.59488186	0.60284297
st dev	0.14102809	0.17738852	0.24039562	0.13893657	0.22160637	0.25248608	0.21750042	0.21740858
t-test of central ring compared to N2 hermaphrodite								
		4.3012E-05	0.0040592	0.00221382	0.23182446	0.00718188	9.0914E-05	7.4828E-05
							t-test with wt male	
								0.93565866

Right X probe

	N2	set-25	lem-2	cec-4	dpy-27(RNAi)
n	12	12	12	12	12
peripheral %	0.25088158	0.3116153	0.25253889	0.27816668	0.11216881
st dev	0.14277669	0.20004045	0.15345298	0.16198887	0.10510497
intermed %	0.54939892	0.48653712	0.61143382	0.55403456	0.58685975
st dev	0.09645279	0.14703048	0.0966098	0.15558981	0.20696769
central %	0.19971952	0.20184757	0.13602729	0.16779875	0.3009714
st dev	0.16839145	0.18234042	0.12730597	0.24469094	0.2564932
t-test of central ring compared to N2 hermaphrodite					
		0.97657508	0.30811983	0.71370518	0.26718607

S1 Table. Statistical analysis of X chromosome FISH with the three zone assay.
n indicates number of nuclei analyzed. Average % of paint signal in each ring and standard deviations are shown.
Results of statistical analysis using Student's test on the portion of the signal in the central ring are below each data set.
