# Supplementary material for: Anchoring of Heterochromatin to the Nuclear Lamina Reinforces Dosage Compensation-Mediated Gene Repression
Source: PLoS Genet. 2016 Sep 30;12(9):e1006341. doi: 10.1371/journal.pgen.1006341 (PMC5045178; doi:10.1371/journal.pgen.1006341)
Supplement: S2 Table — (PDF) [file pgen.1006341.s008.pdf]

Whole chr I paint					
	N2	*met-2*	*set-25*	*cec-4*	*lem-2*
n	10	10	10	10	10
peripheral %	0.27498174	0.28393391	0.32433095	0.27035885	0.33438014
st dev	0.15508742	0.1178846	0.15313704	0.12927037	0.08154668
intermed %	0.32962194	0.40729479	0.42261037	0.38715812	0.37789169
st dev	0.06548054	0.07281563	0.07054663	0.06638567	0.04499781
central %	0.39539632	0.3087713	0.25305867	0.34248303	0.28772817
st dev	0.19366442	0.12723365	0.16203304	0.17888866	0.09688504
t-test of central ring compared to N2 hermaphrodite					
		0.25490246	0.09205845	0.53367557	0.13946616
t-test of central ring compared to X chromosome paint central ring					
	0.03985768				

Left chr I paint		
	N2	*set-25*
n	10	10
peripheral %	0.46685393	0.42616007
st dev	0.17003909	0.15502068
intermed %	0.3625494	0.4885656
st dev	0.10119311	0.1273334
central %	0.17059667	0.08527433
st dev	0.10999698	0.08684983
t-test of central ring compared to N2		
		0.07102638

Middle chr I paint		
	N2	*set-25*
n	10	10
peripheral %	0.12857949	0.13774714
st dev	0.12385027	0.0527957
intermed %	0.51425098	0.46450183
st dev	0.14121135	0.13704882
central %	0.35716953	0.39775103
st dev	0.19140075	0.1152638
t-test of central ring compared to N2		
		0.57436305
t-test of central ring compared to X-mid central ring in N2		
	0.01802029	

Right chr I paint		
	N2	*set-25*
n	10	10
peripheral %	0.48173491	0.46238507
st dev	0.14179603	0.22371649
intermed %	0.4352292	0.40681055
st dev	0.08409888	0.12059219
central %	0.08303589	0.13080437
st dev	0.0771285	0.17828623
t-test of central ring compared to N2		
		0.45154159

S2 Table. Statistical analysis of chromosome I FISH with the three zone assay.
n indicates number of nuclei analyzed. Average % of paint signal in each ring and standard deviations are shown.
Results of statistical analysis using Student's test on the portion of the signal in the central ring are below each data set.
